# Supplementary material for: Policy changes and the screening, diagnosis and treatment of drug-resistant tuberculosis patients from 2015 to 2018 in Zhejiang Province, China: a retrospective cohort study
Source: BMJ Open. 2021 Apr 12;11(4):e047023. doi: 10.1136/bmjopen-2020-047023 (PMC8047997; doi:10.1136/bmjopen-2020-047023)
Supplement: Supplementary data [file bmjopen-2020-047023supp001.pdf]

1. Policy survey questionnaire

Drug-resistant tuberculosis related policy survey in Zhejiang

Prefecture: __________

Institute: __________

Date: _______(YY/MM/DD)

1. Eligibility criteria for drug-resistant tuberculosis (DRTB) patient screening
   1) The current eligibility criteria of patients referred for screening are: ____
   2) Are there any changes in the eligibility criteria after 2014: _____yes/no
      If yes: year of change_______, and the eligibility criteria for patients referred for screening before policy change______________________________________

（If the eligibility criterion is the "high-risk" TB patients, please specify the definition of high-risk TB patients in your prefecture, for example, smear positive after two months' treatment or others）.

2. Test tools for drug susceptibility testing (DST)
   1) Has GeneXpert been equipped in the prefecture-level designated hospital? If yes, it was equipped in the year ______
   2) Under what conditions would patients be referred for traditional DRTB tests after screening? ___________________________
   3) Please list other types of DST conducted in the prefecture, including both molecular and traditional________.
   4) Does the government provide funds to secure the supply of the reagents for rapid DST now? _______(Yes/no). If yes, at which year did the government begin to provide such funds? ____.
3. Financing of DST
   1) Does the patient need to pay out-of-pocket for the DRTB rapid screening test now? _____（yes/no）. If yes, how much does the patient need to pay? RMB_____, accounting for _____% of the total cost
   2) Does the government pay for the DRTB rapid screening test now? （yes/no）. If yes, how much does the government pay for each test? RMB_____, accounting for _____% of the total cost
   3) Does the health insurance pay for the DRTB rapid screening test now? ______（yes/no）. If yes, _____% of the cost would be reimbursed by the health insurance.
   4) Does the health insurance pay for the DRTB rapid screening test now? （yes/no）. If yes, _____% of the cost would be reimbursed by the health insurance.
   5) Have the polices on the financing of DST ever been changed after 2014? If yes, the year of change is ______. Before the policy change,
      the patient paid for RMB____ for the screening test, accounting for _____% of the total cost;
      The government paid for _____% of the total cost;

the health insurance schemes reimbursed _________% of the total cost;

other funding resources paid for ________% of the total cost

4. DRTB patient registration
   1) Currently what types of DRTB patients would be registered and managed in the TB information management system (TBIMS)? (Please check all that apply)

      a. XDR and MDRTB b. rifampin mono-resistant (RMR) patients c. Mono-resistant to other type of first-line drug
   2) Has this policy been changed after 2014? _____(yes/no). If yes, please specify the types of DRTB patients that were registered and under management before policy change. (please check all that apply)

      a. XDR and MDRTB b. rifampin mono-resistant (RMR) patients c. Mono-resistant to other type of first-line drugs
5. DRTB treatment
   1) The standard anti-DRTB treatment regimen includes ______ months' hospitalization, and the total treatment length is _____months
   2) Health insurance policies:
      a) Has the specialized outpatient reimbursement program for DRTB treatment been implemented in your prefecture now? _______(yes/no) If so, this policy began in the year_______. The reimbursement rate for outpatient service is ______%, and the annual limit line for compensation is RMB_______.
      b) The reimbursement rate for DRTB inpatient service is ______%, the deductible is RMB_____, and the annual limit line for compensation is RMB_______.
      c) Has DRTB treatment been included in the serious disease reimbursement program of the health insurance? ________(yes/no). If yes, this program started in the year________, for the out-of-pocket payment over RMB_________, the reimbursement rate is ______%, and the annual limit line is RMB________.
      d) Could RMR patients enjoy the health insurance policies for anti-DRTB treatment now? ________(yes/no). If yes, at which year could the RMR patients start to enjoy such policies? _______.
      e) Are these health insurance policies only eligible for those who enrolled in the local health insurance schemes? _______(yes/no). If no, at which year could patients with health insurance outside your prefecture/Zhejiang province start to enjoy such policies? _______.
      f) Could the health insurance policies be enjoyed if patients do not get cured after the standard treatment length? ______(yes/no). If yes, how long could patients enjoy these policies? ____.
   3) Other government financial assistance policies for DRTB patients
      a) Are there any other financial assistance policies for DRTB patients in your prefecture? _________(yes/no). If yes, at which year were they launched? Please briefly describe the policy? （How much money given to DRTB patients, how to deliver such assistance, etc.）_____________________________________
      b) Could RMR patients enjoy the financial assistance policies for now? _______(yes/no). If yes, at which year could the RMR patients start to enjoy

such policies? _______.

c) Could patients without local registered residence (Hukou) enjoy the policy now? ________(yes/no). If yes, at which year could the RMR patients without local Hukou enjoy such policies? __________.

d) How long could the patients enjoy these policies after diagnosis? ________

6. Any other DRTB related policies, please describe here________________

2. Supplementary result tables

Table 1: Year of policy change regarding the screening test and registration in the 11 prefectures

Prefecture No.	Year of policy change		
	screening range	test technology	registration policy
1	2016	2015	2017
2	2017	2014	2014
3	2017	2017	2017
4	2017	2017	2017
5	2017	2017	2017
6	2009	2016	2019
7	2017	2017	2017
8	2017	2015	2019
9	2017	2017	2017
10	2017	2017	2015
11	2000	2012	2012

Table 2: Starting time and eligibility for health insurance and medical assistance policy coverage, 2015-2018

Prefecture No.	Health insurance policy			Medical assistance		
	starting time	insurance outside ZJ	RMR	starting time	registered residence outside ZJ	RMR
1	2018	N	Y	2013	N	N
2	2013	N	Y	2013	Y	Y since 2015
3	2016	N	N	2014	Y	N
4	2017	N	Y	2015	Y	N
5	2016	N	N	2015	N	N
6	2019	N	N	2012	N	Y
7	2014	N	N	2014	Y	N
8	2013	N	N	2015	N	Y
9	2014	N	Y since 2017	No policy		
10	2013	N	Y	2015	N	Y
11	2011	N	Y	2015	Y	Y

Table 3: The diagnostic test for presumptive DRTB patients from 2015-2018

Year	No. of	% took	% took	% took	Patients with no DST

	presumptive patients reported	fast DST	traditional DST	both tests	total	records (%) not TB	unknown reason
2015	9285	18.2	51.3	0.2	30.7	13.4	17.3
2016	10997	30.4	46.9	0.5	23.3	11.6	11.7
2017	21768	30.9	42.8	5.8	32.2	25.6	6.6
2018	23916	64.4	33.8	20.1	21.9	19.3	2.7

Table 4: Reasons for not completing anti-DRTB treatment according to the registration records

Year	Total	Under treatment		Dead		lost-to-follow-up		Other reasons	
		No.	%	No.	%	No.	%	No.	%
2015	68	11	16.2	17	25.0	13	19.1	27	39.7
2016	101	38	37.6	15	14.9	15	14.9	33	32.7
