# Supplementary material for: Policy changes and the screening, diagnosis and treatment of drug-resistant tuberculosis patients from 2015 to 2018 in Zhejiang Province, China: a retrospective cohort study
Source: BMJ Open. 2021 Apr 12;11(4):e047023. doi: 10.1136/bmjopen-2020-047023 (PMC8047997; doi:10.1136/bmjopen-2020-047023)
Supplement: Author's manuscript [file bmjopen-2020-047023.draft_revisions.pdf]

BMJ Open

BMJ Open is committed to open peer review. As part of this commitment we make the peer review history of every article we publish publicly available.

When an article is published we post the peer reviewers' comments and the authors' responses online. We also post the versions of the paper that were used during peer review. These are the versions that the peer review comments apply to.

The versions of the paper that follow are the versions that were submitted during the peer review process. They are not the versions of record or the final published versions. They should not be cited or distributed as the published version of this manuscript.

BMJ Open is an open access journal and the full, final, typeset and author-corrected version of record of the manuscript is available on our site with no access controls, subscription charges or pay-per-view fees (http://bmjopen.bmj.com).

If you have any questions on BMJ Open's open peer review process please email info.bmjopen@bmj.com

BMJ Open

# Policy changes and the screening, diagnosis and treatment of DRTB patients from 2015-2018 in Zhejiang Province, China: a cascade analysis

| | |
|---|---|
| Journal: | *BMJ Open* |
| Manuscript ID | bmjopen-2020-047023 |
| Article Type: | Original research |
| Date Submitted by the Author: | 18-Nov-2020 |
| Complete List of Authors: | Jiang, Weixi; Duke Kunshan University, Global Health Research Center<br>Peng, Ying; Zhejiang Provincial Center for Disease Control and Prevention<br>Wang, Xiaomeng; Zhejiang Provincial Center for Disease Control and Prevention<br>Elbers, Chris; Vrije Universiteit Amsterdam<br>Tang, Shenglan; Duke University, Duke Global Health Institute<br>Huang, Fei; Centers for Disease Control China, National Center for Tuberculosis Control and Prevention<br>Chen, Bin; Zhejiang Provincial Center for Disease Control and Prevention<br>Cobelens, Frank ; AIGHD, Amsterdam Institute for Global Health and Development and Department of Global Health |
| Keywords: | Tuberculosis < INFECTIOUS DISEASES, Health policy < HEALTH SERVICES ADMINISTRATION & MANAGEMENT, HEALTH SERVICES ADMINISTRATION & MANAGEMENT |
| | |

SCHOLARONE™
Manuscripts

*I, the Submitting Author has the right to grant and does grant on behalf of all authors of the Work (as defined in the below author licence), an exclusive licence and/or a non-exclusive licence for contributions from authors who are: i) UK Crown employees; ii) where BMJ has agreed a CC-BY licence shall apply, and/or iii) in accordance with the terms applicable for US Federal Government officers or employees acting as part of their official duties; on a worldwide, perpetual, irrevocable, royalty-free basis to BMJ Publishing Group Ltd ("BMJ") its licensees and where the relevant Journal is co-owned by BMJ to the co-owners of the Journal, to publish the Work in this journal and any other BMJ products and to exploit all rights, as set out in our licence.*

*The Submitting Author accepts and understands that any supply made under these terms is made by BMJ to the Submitting Author unless you are acting as an employee on behalf of your employer or a postgraduate student of an affiliated institution which is paying any applicable article publishing charge ("APC") for Open Access articles. Where the Submitting Author wishes to make the Work available on an Open Access basis (and intends to pay the relevant APC), the terms of reuse of such Open Access shall be governed by a Creative Commons licence – details of these licences and which Creative Commons licence will apply to this Work are set out in our licence referred to above.*

*Other than as permitted in any relevant BMJ Author's Self Archiving Policies, I confirm this Work has not been accepted for publication elsewhere, is not being considered for publication elsewhere and does not duplicate material already published. I confirm all authors consent to publication of this Work and authorise the granting of this licence.*

For peer review only

**Policy changes and the screening, diagnosis and treatment of DRTB patients from 2015-2018 in Zhejiang Province, China: a cascade analysis**

**Corresponding author:**

**Bin Chen**

Postal address: Zhejiang Provincial Center for Disease Control and Prevention, No. 3399 Binsheng Road, Binjiang District, Hangzhou, China

Email: bchen@cdc.zj.cn

**List of authors:**

**Weixi Jiang**

Global Health Research Center, Duke Kunshan University, Kunshan, Jiangsu, China

Email: weixi.jiang@dukekunshan.edu.cn

**Ying Peng**

Zhejiang Provincial Center for Disease Control and Prevention, Hangzhou, China

Email: ypeng@cdc.zj.cn

**Xiaomeng Wang**

Zhejiang Provincial Center for Disease Control and Prevention, Hangzhou, China

Email: xmwang@cdc.zj.cn

**Chris Elbers**

Faculty of Economics and Business Administration, Vrije Universiteit Amsterdam, Amsterdam, the Netherlands

Email: c.t.m.elbers@vu.nl

**Shenglan Tang**

Duke Global Health Institute, Duke University, Durham, NC, US

Email: shenglan.tang@duke.edu

**Fei Huang**

National Center for Tuberculosis Control and Prevention, China CDC, Beijing, China

Email: huangfei@chinacdc.cn

**Bin Chen**

Zhejiang Provincial Center for Disease Control and Prevention, Hangzhou, China

Email: bchen@cdc.zj.cn

**Frank Cobelens**

The Amsterdam Institute for Global Health and Development, Amsterdam, the Netherlands



For peer review only

For peer review only - http://bmjopen.bmj.com/site/about/guidelines.xhtml

Email: f.cobelens@aighd.org

**Word count: 3781**

For peer review only



For peer review only - http://bmjopen.bmj.com/site/about/guidelines.xhtml

## ABSTRACT

**Introduction**

Drug-resistant Tuberculosis (DRTB) has become a serious concern globally and in China. Several policies have been issued to improve DRTB case finding and treatment. We conducted a cascade analysis on DRTB case finding and care in Zhejiang during 2015-2018 and explored the impacts of these policies.

**Methods**

A questionnaire was designed to undertake a survey of all prefecture-level CDCs in Zhejiang on DRTB-replated policies/programs active or introduced during 2015-2018. De-identified registration data of presumptive and diagnosed DRTB patients from 2015-2018 in Zhejiang were used to construct a seven-step cascade of case finding and care. Mixed-effect two-level logistic regressions were conducted to explore factors associated with patient registration, treatment initiation and treatment completion. The DRTB policies active per prefecture per year were categorized according to the combinations of policies on each step of the cascade, and included in the model to explore their impacts.

**Results**

The number of reported presumptive DRTB patients and the percentage with drug susceptibility testing (DST) records largely increased during 2015-2018, and the percentage of registered patients that received anti-DRTB treatment also increased from 59.0% to 86.5%. Patients under the policies of equipping GeneXpert plus expanded criteria for DST had a higher likelihood of being registered compared with no GeneXpert (adjusted odds ratio, aOR=2.57), while for treatment initiation the association was only when further expanding the registration criteria (aOR=2.38). Patients with registered residence inside Zhejiang were more likely to be registered (aOR=1.96), treated (aOR=3.83) and complete treatment (aOR=1.92) compared with those outside.

**Conclusion**

The policy changes on DST and registration have effectively improved DRTB case finding and care. Nevertheless, challenges remain in servicing the vulnerable group like the migrants and improving equity in the access to TB care. Future policies should provide comprehensive support for migrants to complete treatment at their current place of residence.


**Key words**: Tuberculosis, Health policy



For peer review only - http://bmjopen.bmj.com/site/about/guidelines.xhtml

**Article summary**

**Strengths and limitations of this study**

Strengths

- This study conducted a comprehensive and systematic cascade analysis on the healthcare pathway for drug-resistant tuberculosis (DRTB) patients: screening, diagnosis, treatment and management, and examined key factors associated with patient attrition at the different stages of care, using the TB registration data.
- This study evaluated the effectiveness of different combinations of DRTB related policies in all prefectures of one Chinese province, including new technology promotion, management and financial support, and found that expanding the eligibility criteria of presumptive DRTB patients referred for drug-sensitivity testing (DST), together with the increased funding to guarantee the reagents for rapid assays like GeneXpert, could effectively improve the capacity of DRTB case finding compared with equipping the DST facilities alone.
- This study also examined those patients left-behind during the policy changes from an equity perspective, and found that the inclusion of rifampin mono-resistant (RMR) patients in the registration and management of DRTB patients has closed the management gap between RMR and multidrug-resistant (MDR) TB patients. Nevertheless, formidable challenges remain in reducing inequity in accessing TB care, especially for migrant workers and the elderly due to the lack of appropriate supporting policies for these patient groups to obtain adequate health services.

Limitations

- Almost all prefectures had some form of financing policies since 2015 and there were no individual-level data on whether the patient benefited from these financing policies. Therefore, in this study the impacts of these financing policies could not be explored.
- Other potentially important socio-economic factors like education and income were not available in the dataset used, limiting therefore the scope of the equity analysis to migrant workers.

For peer review only



For peer review only - http://bmjopen.bmj.com/site/about/guidelines.xhtml

## INTRODUCTION

Drug-resistant tuberculosis (DRTB) has become a global concern in recent years. It is estimated that globally in 2018, 3.4% of new TB patients and 18% of previously treated patients developed rifampicin-resistant forms of disease (RRTB) including multi-drug resistant TB (MDR-TB), in which there is additional resistance to isoniazid, and 7.1% and 21% respectively in China.[1] Currently China accounts for 14% of the RR/MDR-TB disease burden, and one recent projection suggested the incidence of RR/MDR-TB would triple without interventions to change current conditions.[2] The RR/MDR-TB epidemic has posed a great challenge to achieve the SDG target of ending TB in 2030 in China.[3]

The diagnosis and treatment of DRTB can be very costly in terms of both time and money,[4] and barriers to accessing DRTB diagnosis and care exist worldwide.[5] The diagnosis of DRTB takes 1-3 months using traditional technology, and the treatment of RR/MDR-TB lasts for up to two years. DRTB patients generally have lower socio-economic status, and the cost of treatment is so high that the current financing policies in the form of health insurance reimbursement and subsidized treatment are far from sufficient.[6-8] For China specifically, the public health insurance programs provide very limited coverage for outpatient services that are required for around 20 months for RR/MDR-TB patients, and some second-line anti-TB drugs and auxiliary drugs are often not covered.[9-11] Previous studies have also revealed the long treatment delay, the high pre-diagnosis, pre-treatment attrition as well as loss-to-follow-up during the treatment course in several countries including China.[12-17] Moreover, the MDR-TB treatment success rate in China was less than 50%.[18]

This formidable barriers for DRTB patients to accessing and adhering to standard treatment call for

For peer review only

strong supporting policies for patients to receive and complete treatment.[19] Previous studies have validated the utility of rapid drug susceptibility testing (DST) technologies such as Genechip and GeneXpert in the screening for DRTB, and showed that they could improve DRTB case finding, shorten treatment delays and decrease pre-diagnosis attrition.[13, 20–24] As for treatment adherence, one comprehensive program in China that provided universal health coverage to MDR-TB patients was shown to improve access to and affordability of diagnosis and quality treatment of MDRTB.[5] Studies on other intervention strategies showed that directly observed therapy (DOT) or other reminding approaches through digital technologies could improve the treatment adherence and outcomes of TB/MDRTB.[25, 26] Patient counseling alone or combined with financial support could also increase the cure rates among MDRTB patients.[27]

In China some provinces, along with or after the roll-out of international donor-funded projects, have implemented policies to improve DRTB control, including allocating special funds to equip DRTB-designated hospitals with DST facilities and reagents, improving health insurance benefit packages and providing subsidies to patients.[11,28] However, there is limited evidence on the impact of implementing these policies on DRTB case finding and care thereafter. The equity issues underlying the case finding and care procedures for DRTB patients are also understudied, especially considering that the eligibility for policies issued in a certain region is often linked with patients' registered residence, work and health insurance status.

This study aims to examine changes in the programmatic performance with regard to screening, diagnosis, treatment and management of DRTB patients in Zhejiang from 2015 to 2018 through the cascade analysis approach.[9] Besides, as the policies and guidelines on this whole procedure of DRTB care changed during this period, this study systematically summarizes these changes and



For peer review only - http://bmjopen.bmj.com/site/about/guidelines.xhtml

investigates how these policy changes have influenced case finding and treatment of DRTB patients. We also explore whether these policy changes have equally influenced patients of different demographic and socio-economic characteristics.

## METHODS

### Study settings

Zhejiang is a province located in the eastern area with its GDP ranking 4th in China,[30] and has a growing migrant population in recent years. Under the current TB control model in Zhejiang, the prefecture-level designated hospital, usually one in each prefecture, is responsible for the diagnosis and treatment of DRTB patients, while the Center for Disease Control (CDC) and primary health care facilities conduct patient management. Patients who are clinically suspected to have DRTB are to be referred for DST and should be reported in the Tuberculosis Information Management System (TBIMS) as presumptive DRTB patients. Their sputum samples are to be sent to the prefecture-level designated hospital for DST. If diagnosed with DRTB, these patients should be registered in the TBIMS database for diagnosed patients as such. The criteria regarding what types of DRTB should be registered have being changing over time.

### Study Design

This study included a questionnaire survey on the DRTB policies/programs among the 11 prefecture CDCs and a quantitative analysis of the TBIMS records of presumptive and diagnosed DRTB patients.

Questionnaire survey on DRTB policies/programs



For peer review only - http://bmjopen.bmj.com/site/about/guidelines.xhtml

For peer review only

A questionnaire on the DRTB related policies was distributed to the CDC of all prefectures in Zhejiang in collaboration with Zhejiang provincial CDC. After preliminary consultation with the provincial CDC, the questionnaire was designed to include policies in four areas: 1) eligibility of presumptive DRTB patients for DST, 2) eligibility for registering DRTB patients based on the drug-resistant type (including rifampin mono-resistance (RMR), multi-drug resistance(MDR), extensive drug-resistance (XDR) and mono-resistance to other types of drugs), 3) DST technology and payment for DST and 4) financing policies, including both health insurance benefit packages and government subsidies for supporting the DRTB treatment. Detailed questions on the eligibility for financial support regarding the types of DRTB, the registered permanent residence and the region of health insurance enrollment were also included in the questionnaire. If the policies changed at any time after 2015, we collected information on the policy details before and after change as well as the year of change.

TBIMS records

De-identified TBIMS records of presumptive and diagnosed DRTB patients from 2015-2018 in Zhejiang were retrieved from the TB Prevention and Control Center of China CDC. The dataset of presumptive DRTB patients included demographic information (prefecture of registration, age, sex, ethnicity, occupation and registered residence), drug-resistance test profile (the date of sending the sample, conducting the test and reporting the result, the type of test and the test result), as well as a unique registration number if registered as a DRTB patient in the diagnosed patient dataset. Only those DRTB patients recorded in the diagnosed patient dataset have a trackable treatment history and are managed under a specialized guideline for DRTB patients. The dataset of diagnosed DRTB patients contained the same demographic information and diagnostic data as the dataset of



For peer review only - http://bmjopen.bmj.com/site/about/guidelines.xhtml

presumptive patients, plus the treatment information including the starting date of anti-DRTB treatment including both second-line (RR/MDR-TB) and adapted first-line (monoresistance to isoniazid) treatment, TB treatment history, current state of treatment (under treatment or ended), and the ending date of treatment as well as the reason of ending the treatment (cured/treatment completed/death/lost-to-follow-up/others) if ended. In TBIMS the treatment status referred to whether the patient received treatment in Zhejiang. If the patients were registered but returned to their hometown for treatment, it could be shown as "treatment refused".

**Patient and Public Involvement Statement**

Patients or the public were not involved in the design, or conduct, or reporting, or dissemination plans of our research.

**Data analysis**

Using datasets of the presumptive and registered diagnosed patients, a seven-step cascade of diagnosis and care was constructed for each year from 2015 to 2018 : 1) reported presumptive DRTB patients; 2) presumptive patients who had DST; 3) diagnosed DRTB, and RR/MDRTB (including RMR, MDR and XDR) patients; 4) registered RR/MDRTB patients; 5) RR/MDRTB patients that ever received anti-DRTB treatment; 6) RR/MDRTB patients that were under treatment 6 months after treatment initiated; 7) RR/MDRTB patients that had been cured or completed treatment, as shown in Figure 1. Diagnosed DRTB patients were defined as patients with a DST result showing resistance to at least one type of first-line anti-TB drug. As in all but one prefectures only RR/MDRTB patients are required to be registered and thus had available data on treatment history



For peer review only - http://bmjopen.bmj.com/site/about/guidelines.xhtml

before 2019, we only analyzed RR/MDRTB patients for step 4-7. Descriptive analyses were conducted to explore the reasons for attrition at each step based on the relevant records in the datasets. Frequencies of patient characteristics were also calculated across patient groups at each step of the cascade to examine potential factors associated with the attrition at different steps. For step 3-5 frequencies of some patient characteristics of interests, including age, sex, drug-resistant type, occupation and registered residence were calculated separately for each year to examine the trends from 2015 to 2018.

Mixed-effect two-level logistic regressions, with fixed slope and random intercept specification were conducted to explore factors associated with the likelihood of being registered for RRTB patients, the likelihood of receiving anti-DRTB treatment if registered, and the likelihood of getting cured or completing treatment (the latter only for those who initiated treatment before 2017 because of the 2-year treatment duration). This model showed better fit to the data than the fixed-effect model based on the likelihood-ratio test. The DRTB related policies were summarized along the four areas in the questionnaires. Besides patient-level variables, the policies implemented in each prefecture and each year, categorized according to the different combinations of policies in the four areas, were included in the model. Per-capita GDP of the prefecture were divided into three groups, as the numbers assembled in three intervals: RMB 55,000-70,000, 80,000-100,000, and above 120,000, and also included in the model as a categoric variable. The clustered standard errors were estimated considering the intra-prefecture correlations.

## RESULTS

### DRTB policy change from 2015-2018 in Zhejiang



For peer review only - http://bmjopen.bmj.com/site/about/guidelines.xhtml

Results from the questionnaire survey on DRTB policies showed that the major change in the eligibility of patients for DST was the expansion from high-risk patients (including smear positive relapse, treatment failure, positive sputum bacteriology after two months' treatment), to all bacteriologically positive TB patients. As for the testing technology, the major change was the introduction of the rapid DST assay GeneXpert MTB/RIF that combines testing for Mycobacterium tuberculosis with screening for rifampicin-resistance with a same-day result, while the costs for the test were all covered, or mostly covered by the government. The criteria for DRTB patient registration (implying eligibility for specialized DRTB patient management) also expanded from MDR/XDR-TB to RR-TB, and one prefecture even expanded to any DRTB before 2018. In most prefectures these changes in DST eligibility and equipment as well as the registration criteria happened in 2017, while almost all others had adopted the new polices and introduced GeneXpert earlier (supplemental table 1).

The financing policies to support DRTB treatment in Zhejiang consisted of both health insurance and medical assistance policies, and in most prefectures these policies had been implemented in 2015. These financing policies aimed to improve the benefit package for DRTB patients through a three-layer coverage system: 1) increasing the reimbursement rate of outpatient treatment to 70%-90%, the same level as for inpatient treatment; 2) the out-of-pocket (OOP) expenditure exceeding a certain amount, typically RMB 20,000-30,000, could be further reimbursed at a rate of 55%-85% through a supplemental health insurance program for critical illnesses; 3) the OOP expenditure after health insurance reimbursement could be covered by the medical assistance, with a payment limit varying from 11,000 RMB to over 60,000 RMB across prefectures. As for the eligibility for the insurance policies, those with public health insurance enrolled outside Zhejiang, usually migrants



For peer review only - http://bmjopen.bmj.com/site/about/guidelines.xhtml

without formal jobs, were excluded. In 5 out of the 11 prefectures RMR patients were still not eligible for the expanded public health insurance benefit package of up to 2019. For the medical assistance, in 6 out of the 11 prefectures only patients with registered residence ("Hukou") in Zhejiang were eligible, and for the other 4 prefectures with assistance policies the eligibility had been expanded to all residents in this prefecture. Up to 2018, RMR patients in 5 prefectures were still excluded from the medical assistance (supplemental table 2).

**Cascade analysis of DRTB care from 2015-2018**

Table 1 shows the screening and diagnosis of DRTB patients from 2015-2018. In general, the results suggested increased capacity to find DRTB patients. The number of reported presumptive DRTB patients increased from 9285 to 23916, with the largest increase from 2016 to 2017 coinciding with the change in screening policy in most prefectures. The percentage of patients with test results also increased from 69.3% to 78.1%. Along with the increase in the volume of DST there was an increase in the number of diagnosed DRTB patients except for 2018, and a decrease in the percentage of patients diagnosed among those tested. RR/MDR-TB patients accounted for around 40%-50% of the total DRTB patients.

Further analysis on the types of diagnostic tests patients received revealed an increasing trend in the percentage of patients taking rapid DST as well as the percentage of patients tested with both conventional culture-based and rapid tests. The percentage of presumptive patients without DST profile for unknown reasons as recorded in the TBIMS dataset, which indicates pre-diagnosis attrition, dropped dramatically from 17.3% to 2.7% (supplemental table 3).



For peer review only

Table 1: Diagnostic cascade starting from presumptive DRTB patients.

| Year | No. of presumptive DRTB patients | Patients with test records | | Diagnosed DRTB patients | | Diagnosed RR/MDR-TB patients | |
|---|---|---|---|---|---|---|---|
| | | No. | % | No. | % | No. | % |
| 2015 | 9285 | 6434 | 69.3 | 1031 | 16.0 | 503 | 48.8 |
| 2016 | 10997 | 8438 | 76.7 | 1258 | 14.9 | 529 | 42.1 |
| 2017 | 21768 | 14764 | 67.8 | 1729 | 11.7 | 716 | 41.4 |
| 2018 | 23916 | 18670 | 78.1 | 1580 | 8.5 | 663 | 42.0 |

Table 2 shows the registration and treatment for diagnosed RRTB patients from 2015-2018. While the actual number of registered RRTB patients increased, the percentage of registered RRTB patients dropped in 2017, and this percentage increased again to 84.6%. The percentage of registered patients that received anti-DRTB treatment increased from 59.0% to 86.5%, and the percentage of those treated who received at least 6 month's treatment remained above 90%. Around 70% of patients diagnosed in 2015 and 2016 completed treatment. Duration of treatment could be longer than 2 years, as 38 out of the 283 patients starting treatment in 2016 were still shown as under treatment at the time we retrieved the data (see supplemental table 4).

Table 2: Registration and treatment cascade for diagnosed RRTB patients.

| Year | Diagnosed RR/MDR-TB patients | Registered | | Received anti-DRTB treatment | | Under treatment 6 months after treatment initiated | | Cured or completed treatment | |
|---|---|---|---|---|---|---|---|---|---|
| | | No. | % | No. | % | No. | % | No. | % |
| 2015 | 503 | 383 | 76.1 | 226 | 59.0 | 215 | 95.1 | 158 | 73.5 |
| 2016 | 529 | 410 | 77.5 | 283 | 69.0 | 263 | 92.9 | 182 | 69.2 |
| 2017 | 716 | 502 | 70.1 | 360 | 71.7 | 334 | 92.8 | / | / |
| 2018 | 663 | 561 | 84.6 | 485 | 86.5 | 462 | 95.3 | / | / |

**Factors associated with the registration and treatment of DRTB patients**

Table 3 shows the characteristics of patients across each step of the care cascade. Around 70% of



For peer review only - http://bmjopen.bmj.com/site/about/guidelines.xhtml

the presumptive and diagnosed patients were male, and around 98% of them were Han People. Only around 40% of the patients had formal jobs other than farming or unemployment. While under the assumption of perfect equity we would expect the percentage of patients with different characteristics to remain the same from the diagnosed to the treated group, it decreased from 30.3% to 27.0% for older patients, 28.1% to 22.5% for RMR patients, and from 24.4 to 17.0% for patients with registered residence outside Zhejiang, i.e., migrants. An increase was observed regarding the percentage of patients ever taking rapid DST.

Table 3: Patient characteristics across each step of the care cascade

| | Presumptive DRTB patients | Diagnosed DRTB patients | Diagnosed RR/MDR-TB patients | Registered RR/MDR-TB patients | RR/MDR-TB patients recevied treatment |
|---|---|---|---|---|---|
| N | 65966 | 5598 | 2411 | 1859 | 1357 |
| Age (%) | | | | | |
| >=60 | 41.3 | 35.9 | 30.3 | 29.8 | 27.0 |
| Gender (%) | | | | | |
| Male | 70.6 | 73.6 | 72.5 | 72.7 | 72.7 |
| Ethnicity | | | | | |
| Han (%) | 97.7 | 98.0 | 97.9 | 98.0 | 98.8 |
| Patient type (%) | | | | | |
| new patient | 70.2 | 65.2 | 51.8 | 51.4 | 48.8 |
| Drug-resistanct type (%) | | | | | |
| RMR | / | 12.1 | 28.1 | 20.8 | 22.5 |
| MDR | / | 30.4 | 70.6 | 78.0 | 76.1 |
| XDR | / | 0.5 | 1.2 | 1.2 | 1.4 |
| Non-rifampicin resistant | / | 56.9 | / | / | / |
| Registered residence (%) | | | | | |
| outside Zhejiang | 23.4 | 25.2 | 24.4 | 22.3 | 17.0 |
| Job category (%) | | | | | |
| farmers | 49.0 | 48.0 | 43.8 | 45.0 | 45.5 |
| unemployed | 12.4 | 13.0 | 14.1 | 14.2 | 14.0 |
| other | 38.6 | 39.0 | 42.1 | 40.8 | 40.5 |
| Test type (%) | | | | | |
| fast test | / | 38.6 | 46.5 | 46.1 | 49.2 |



For peer review only - http://bmjopen.bmj.com/site/about/guidelines.xhtml

Figure 2 shows the changes in the characteristics of diagnosed, registered and treated RR/MDR-TB patients from 2015-2018. The percentage of diagnosed RMR patients that received adequate treatment was low in 2015 and 2016, but increased dramatically from 2017, coinciding with the policy change. The gaps between the proportion of older patients in the diagnosed and the treated group also narrowed. Nevertheless, over the four years there remained a 7-10 percentage decrease in the proportion of patients with registered residence outside Zhejiang from the diagnosed to the treated patient group, indicating that migrants were still more likely to drop out after diagnosis compared with the local residents. Gender and job category were not obviously associated with the pre-treatment attrition in this univariate analysis.

To systematically investigate the impact of policies on DRTB case finding and care, we divided the combination of policies in different areas of DRTB implemented in each prefecture for each year during 2015-2018 into four categories, considering both policy implications and the number of cases in each category. As shown in Figure 3, since 2017 all prefectures had equipped GeneXpert and expanded the criteria for DST as well as registration, except for two prefectures which still did not register RMR patients.

Table 4 shows the results of mixed-effect two-level logistic regression of the factors associated with registration among diagnosed RRTB patients, as well as with receiving anti-DRTB treatment among those registered. After adjusting for other factors, patients under the policy of providing GeneXpert together with expanding eligibility for DST (category 3, adjusted odds ratio (aOR) =2.57, 95% CI: 1.20, 5.51) had a significantly higher likelihood of being registered compared to patients not being provided GeneXpert, while this association was not significant for providing GeneXpert testing without expanding the eligibility (category 2). A positive association with receiving anti-DRTB



For peer review only

treatment was significant only for the policy combination of providing GeneXpert and expanding eligibility for both DST and registration (category 4, aOR=2.38, 95%CI: 1.19, 4.79). Not surprisingly, MDR/XDR-TB patients were much more likely to be registered compared with RMR patients due to the registration policy in earlier time periods. In addition, patients with registered residence inside Zhejiang were more likely to be registered (aOR=1.96, 95% CI 1.52, 2.52) or treated (aOR=3.83, 95% CI 2.78, 5.28). Older age was associated with lower likelihood both of being registered (aOR=0.69,95% CI 0.53, 0.90) and of receiving anti-DRTB treatment (aOR=0.40, 95% CI 0.30, 0.52).

Tables 4: Factors associated with registration for diagnosed RRTB patients, and with receiving anti-DRTB treatment for registered RRTB patients.

| | Registration (n=2367) | | | | Receiving treatment (n=1824) | | | |
|---|---|---|---|---|---|---|---|---|
| | OR | P>z | 95% CI | | OR | P>z | 95%CI | |
| Age | | | | | | | | |
| >=60 | 0.69 | 0.006 | 0.53 | 0.90 | 0.40 | 0.000 | 0.30 | 0.52 |
| Gender | | | | | | | | |
| male | 1.08 | 0.554 | 0.85 | 1.37 | 0.87 | 0.425 | 0.62 | 1.22 |
| DR type | | | | | | | | |
| MDR/XDR-TB | 5.93 | 0.000 | 3.10 | 11.36 | 0.91 | 0.789 | 0.45 | 1.84 |
| Patient type | | | | | | | | |
| new patient | 1.22 | 0.146 | 0.93 | 1.59 | 0.52 | 0.000 | 0.37 | 0.72 |
| Treatment history | NA | | | | | | | |
| no treatment | | | | | ref. | | | |
| 1st-line drug only | | | | | 0.58 | 0.044 | 0.34 | 0.99 |
| 2nd-line drug used | | | | | 1.11 | 0.748 | 0.58 | 2.12 |
| Policy category | | | | | | | | |
| 1 | ref. | | | | ref. | | | |
| 2 | 1.18 | 0.626 | 0.61 | 2.29 | 0.87 | 0.776 | 0.34 | 2.25 |
| 3 | 2.57 | 0.015 | 1.20 | 5.51 | 1.44 | 0.257 | 0.77 | 2.68 |
| 4 | 2.08 | 0.054 | 0.99 | 4.37 | 2.38 | 0.015 | 1.19 | 4.79 |
| Registered residence | | | | | | | | |
| in Zhejiang | 1.96 | 0.000 | 1.52 | 2.52 | 3.83 | 0.000 | 2.78 | 5.28 |
| Job category | | | | | | | | |
| farmers | ref. | | | | ref. | | | |
| unemployed | 1.21 | 0.017 | 1.04 | 1.42 | 0.90 | 0.651 | 0.58 | 1.40 |



For peer review only

For peer review only - http://bmjopen.bmj.com/site/about/guidelines.xhtml

| | | | | | | | | |
|---|---|---|---|---|---|---|---|---|
| other jobs | 0.92 | 0.281 | 0.80 | 1.07 | 0.94 | 0.784 | 0.60 | 1.47 |
| Per capita GDP | | | | | | | | |
| highest group | ref. | | | | ref. | | | |
| middle group | 1.72 | 0.122 | 0.87 | 3.42 | 2.20 | 0.025 | 1.10 | 4.38 |
| lowest group | 1.70 | 0.099 | 0.90 | 3.20 | 1.15 | 0.643 | 0.63 | 2.12 |
| _cons | 0.32 | 0.002 | 0.16 | 0.66 | 1.85 | 0.207 | 0.71 | 4.78 |
| prefecture var(_cons) | 0.18 | | 0.09 | 0.39 | 0.18 | | 0.03 | 0.92 |

Tables 5 shows the results of the mixed-effect two-level logistic regression analysis of factors associated with the likelihood of completing treatment for patients who initiated treatment before 2017. All prefectures had some kind of financing policies in 2015 and 2016, and most prefectures had not yet expanded the eligibility of patients for DST or registration. We therefore used the individual level data on utilization of rapid DST to assess the impact of rapid testing on treatment completion. After adjusting for other factors, older patients were less likely to complete treatment (aOR=0.24, P<0.001), while registered residence inside Zhejiang was associated with higher probability of completing treatment (aOR=1.92, P<0.05). Utilization of rapid DST was not associated with treatment completion. These two factors had significant impacts of the same direction on the registration, treatment initiation and treatment completion of RR/MDRTB patients.

Table 5: Factors associated with the likelihood of completing treatment for patients that initiated treatment before 2017

| | OR | P>z | 95% Conf. | Interval |
|---|---|---|---|---|
| Age | | | | |
| >=60 | 0.24 | <0.001 | 0.14 | 0.42 |
| Gender | | | | |
| male | 0.79 | 0.442 | 0.44 | 1.44 |
| Drug-resistance type | | | | |
| MDR/XDR-TB | 0.50 | 0.086 | 0.23 | 1.10 |
| Patient type | | | | |
| new patient | 1.06 | 0.820 | 0.64 | 1.77 |
| TB treatment history | | | | |
| no treatment history | ref. | | | |
| first-line drug only | 0.74 | 0.355 | 0.39 | 1.40 |



For peer review only - http://bmjopen.bmj.com/site/about/guidelines.xhtml

| | | | | |
|---|---|---|---|---|
| second-line drug used | 1.07 | 0.847 | 0.56 | 2.02 |
| Test type | | | | |
| fast test | 0.70 | 0.096 | 0.46 | 1.06 |
| Registered residence | | | | |
| in Zhejiang | 1.92 | 0.040 | 1.03 | 3.59 |
| Job category | | | | |
| farmers | ref. | | | |
| unemployed | 0.84 | 0.635 | 0.42 | 1.71 |
| other jobs | 1.44 | 0.278 | 0.74 | 2.79 |
| Per capita GDP | | | | |
| highest group | ref. | | | |
| middle group | 0.94 | 0.939 | 0.20 | 4.39 |
| lowest group | 1.07 | 0.921 | 0.30 | 3.74 |
| Year of sending sample | | | | |
| 2015 | ref. | | | |
| 2016 | 1.23 | 0.164 | 0.92 | 1.64 |
| _cons | 5.58 | 0.001 | 2.01 | 15.50 |
| prefecture var(_cons) | 0.21 | | 0.04 | 1.10 |

## DISCUSSION

Results from this study clearly revealed the positive impacts of the combined policy change regarding the DST of presumptive DRTB patients and the registration of diagnosed patients. However, inequity challenges remain in terms of servicing vulnerable groups, e.g. migrant workers and the older population, in the registration, treatment and management of DRTB.

### Effectiveness and challenges of the policy interventions

The expansion of the eligibility criteria of presumptive DRTB patients referred for DST, together with the increased funding support to equip the facilities and guarantee the reagents for rapid DST like GeneXpert, have largely improved the capacity for DRTB case finding. The significant effects of the combined policies compared to providing GeneXpert alone indicates that in order to effectively improve case finding we need to not just introduce new technologies, but also support



For peer review only - http://bmjopen.bmj.com/site/about/guidelines.xhtml

and expand their use. The inclusion of RMR in the registration and management of DRTB patients closed the management gap between RMR and MDR/XDR-TB patients, and also improved the treatment rate for RR/MDR-TB.

It is obvious that these reforms in the DRTB policies and regulations have effectively changed the practices in DRTB control. Nevertheless, no policies aiming at migrants were issued between 2015 to 2018, and during these four years the degree of inequity between local and migrant patients did not seem to be mitigated either. The improved insurance benefit package was only available for those with public health insurance enrollment in Zhejiang, and in many prefectures receiving medical assistance still required local registered residence during 2015-2018. Besides, older people were less likely to be registered or receive treatment. This may be because they were more likely to give up treatment due to the high cost and long course of the standard treatment, and health workers may not register them once they refused to provide information necessary for registration. These findings were consistent with previous studies that age and migration for work, as well as health system factors such as lack of clear eligibility criteria for DST and limited capacity to provide DST were associated with attrition at different stages of the cascade.[14, 16, 31–33] Nevertheless, no impacts were observed of other factors, such as association between treatment experience and treatment outcome, and data on other socio-economic factors such as financial difficulties were not available in this study.

**Equity challenges regarding migrant populations**

The growing number of migrants in China and other parts of the world are posing a challenge to TB control.[31, 34–36] Though one systematic review found no significant differences in treatment



For peer review only - http://bmjopen.bmj.com/site/about/guidelines.xhtml

adherence between migrant and long-term resident MDRTB patients,[37] several studies identified barriers for migrants to accessing TB diagnosis and care mainly in terms of knowledge gaps and financial difficulty,[38, 39] and these barriers would likely to be more substantial for migrant MDRTB patients. In our study, only around 40% of the DRTB patients had a formal job other than farmers or self-employment. In China people with a formal job will be compulsively enrolled in the urban employee basic medical insurance at the place of work as required by the law. However, migrants without a formal job often choose to participate in the health insurance for residents in their hometown which requires lower premium compared to the more developed region they migrated to, and thus cannot benefit from the reimbursement policy in their working place. Besides, they often have to pay the full cost of treatment first before they get reimbursed when they return home. Therefore, it is expected that many of these DRTB patients would choose to go back to their hometown for treatment, or even refuse treatment. The need for migrant DRTB patients to travel long-distances and the substandard or even absent treatment received would increase the risk of disease transmission and treatment failure. A study in Shanghai, a well-developed city in eastern China, showed that financial incentives were effective for migrant TB patients to complete treatment.[40] In terms of these findings, policies aimed at encouraging migrant DRTB patients to be treated and managed at their current place of residence need to be developed, and this is particularly urgent for regions with a large number of migrant populations.

One major limitation of the study is that almost all prefectures had some form of financing policy since 2015 and there were no individual-level data on whether the patient benefited from these financing policies. Therefore, in this study the impacts of these financing policies could not be explored. Furthermore, other potentially important socio-economic factors like education and



For peer review only - http://bmjopen.bmj.com/site/about/guidelines.xhtml

income were not available in our dataset, limiting the scope of the equity analysis. Nevertheless, these limitations would not influence our conclusions that changes in the screening and registration of DRTB patients have largely increased the case finding and management capacity of DRTB patients, while during the period 2015-2018 differences between the local and migrant patients in the registration, treatment initiation and treatment outcome remained. Future research should collect more individual-level data on the implementation of the health insurance and medical assistance policies to investigate the impacts of these demand-side policies on DRTB treatment more directly, and explore what kind of financing policies provided for migrants could increase their likelihood of initiating and completing anti-DRTB treatment.

## DECALRATIONS

### Ethical approval

This study is under the overall evaluation study of China-Gates TB Project Phase III and used data collected from this project. The implementation of this project has received the ethical approval from China CDC (No.201626). The protocol of the overall study design and data collection tools was reviewed and approved by the Institutional Review Board of Duke University (IRB Approval code: 2017-0768). This paper does not use data involving human participants.

### Competing interests

All authors declare no competing interests.

### Funding

The work was supported by the Bill and Melinda Gates Foundation grant number [OPP1149395].

### Contributorship statement

The study was designed by Weixi Jiang, Shenglan Tang, Chris Elbers and Frank Cobelens. Fei Huang, Ying Peng, Xiaomeng Wang, Bin Chen and Weixi Jiang coordinated and conducted data collection. Weixi Jiang conducted literature review and wrote the manuscript as the first author. Shenglan Tang, Chris Elbers, Bin Chen and Frank Cobelens provided suggestions on data analysis framework and data interpretation, and also revised the manuscript. All authors reviewed the draft manuscript and provided comments on the finalization of the manuscript. All authors have read and approved the manuscript in its current state.

### Data sharing statement

No additional data available.



For peer review only - http://bmjopen.bmj.com/site/about/guidelines.xhtml

**Acknowledgements**

This paper is part of the outputs emanating from the program entitled "China National Health and Family Planning Commission and the Gates Foundation TB Project (Phase III)"-a collaboration between the Government of China and the Melinda and Bill Gates Foundation, and implemented by the China Center of Disease Control and Prevention (CDC). The authors of the paper also gratefully acknowledge the officers in Zhejiang Provincial CDC and the 11 prefectural CDCs for helping with collecting DRTB related policies in Zhejiang.

**REFERENCES**

1. World Health Organization. Global Tuberculosis Report 2019: P58
2. Li BY, Shi WP, Zhou CM, et al. Rising challenge of multidrug-resistant tuberculosis in China: A predictive study using Markov modeling. *Infect Dis Poverty.* 2020;9(1):1-8. doi:10.1186/s40249-020-00682-7
3. World Health Organization. accessed August, 2020 https://www.who.int/sdg/targets/en/
4. Fitzpatrick C, Floyd K. A Systematic Review of the Cost and Cost Effectiveness of Treatment for Multidrug-Resistant Tuberculosis. *Pharmacoeconomics*. 2012;30(1):63-80. doi:10.2165/11598640-000000000-00000
5. Li R, Ruan Y, Sun Q, et al. Effect of a comprehensive programme to provide universal access to care for sputum-smear-positive multidrug-resistant tuberculosis in China: A before-and-after study. *Lancet Glob Heal.* 2015;3(4):e217-e228. doi:10.1016/S2214-109X(15)70021-5
6. Long Q, Qu Y, Lucas H. Drug-resistant tuberculosis control in China: progress and challenges. *Infect Dis Poverty*. 2016;5(1):9. doi:10.1186/s40249-016-0103-3
7. Hutchison C, Khan MS, Yoong J, Lin X, Coker RJ. Financial barriers and coping strategies: a qualitative study of accessing multidrug-resistant tuberculosis and tuberculosis care in Yunnan, China. *BMC Public Health*. 2017;17(1):1-11. doi:10.1186/s12889-017-4089-y
8. Wingfield T, Boccia D, Tovar M, et al. Defining Catastrophic Costs and Comparing Their Importance for Adverse Tuberculosis Outcome with Multi-Drug Resistance: A Prospective Cohort Study, Peru. *PLoS Med*. 2014;11(7). doi:10.1371/journal.pmed.1001675
9. Pan H-Q, Bele S, Feng Y, et al. Analysis of the economic burden of diagnosis and treatment of tuberculosis patients in rural China. *Int J Tuberc Lung Dis.* 2013;17(12):1575-1580. doi:10.5588/ijtld.13.0144
10. Wang Y, McNeil EB, Huang Z, et al. Household financial burden among multidrug-resistant tuberculosis patients in Guizhou province, China: A cross-sectional study. *Med.* 2020;99(28):e21023. doi:10.1097/MD.0000000000021023
11. Tang S, Wang L, Wang H, Chin DP. Access to and affordability of healthcare for TB patients in China: Issues and challenges. *Infect Dis Poverty.* 2016;5(1):1-5. doi:10.1186/s40249-016-0096-y
12. Hemant Deepak Shewade, Dina Nair, Joel S. Klinton, et al. Pre-diagnosis attrition but high pre-treatment attrition among patients with MDR-TB: An operational research from Chennai,India. *J Epidemiol Glob Health*. 2017;7(4):227-233.
13. Boyd R, Ford N, Padgen P, Cox H. Time to treatment for rifampicin-resistant tuberculosis: Systematic review and meta-analysis. *Int J Tuberc Lung Dis.* 2017;21(11):1173-1180.



For peer review only - http://bmjopen.bmj.com/site/about/guidelines.xhtml

doi:10.5588/ijtld.17.0230

14. Xu C, Li R, Shewade HD, et al. Attrition and delays before treatment initiation among patients with MDR-TB in China (2006-13): Magnitude and risk factors. *PLoS One*. 2019;14(4):1-16. doi:10.1371/journal.pone.0214943
15. Hirasen K, Berhanu R, Evans D, et al. High rates of death and loss to follow-up by 12 months of rifampicin resistant TB treatment in South Africa. *PLoS One*. 2018;13(10):1-14. doi:10.1371/journal.pone.0205463
16. Shewade D, Kokane AM, Singh AR, et al. High pre-diagnosis attrition among patients with presumptive MDR-TB: an operational research from Bhopal district, India. *BMC Health Serv Res*. 2017;17(1):1-10. doi:10.1186/s12913-017-2191-6
17. Oo T, Kyaw KWY, Soe KT, et al. Magnitude and reasons for pre-diagnosis attrition among presumptive multi-drug resistant tuberculosis patients in Bago Region, Myanmar: A mixed methods study. *Sci Rep*. 2019;9(1):1-10. doi:10.1038/s41598-019-43562-3
18. Xu C, Pang Y, Li R, et al. Clinical outcome of multidrug-resistant tuberculosis patients receiving standardized second-line treatment regimen in China. *J Infect.* 2018;76(4):348-353. doi:10.1016/j.jinf.2017.12.017
19. Abubakar I, Zignol M, Falzon D, et al. Drug-resistant tuberculosis: Time for visionary political leadership. *Lancet Infect Dis*. 2013;13(6):529-539. doi:10.1016/S1473-3099(13)70030-6
20. Radin E, Ariana P, Broekel T et al. Analyzing demand-side efficiency in global health: an application to maternal care in Vietnam. *Health Policy Plan.* 2016;31(9):1281-1290. doi:10.1093/heapol/czw063
21. Boehme CC, Nicol MP, Nabeta P, et al. Feasibility, diagnostic accuracy, and effectiveness of decentralised use of the Xpert MTB/RIF test for diagnosis of tuberculosis and multidrug resistance: A multicentre implementation study. *Lancet.* 2011;377(9776):1495-1505. doi:10.1016/S0140-6736(11)60438-8
22. Christopher PM, Cucunawangsih, Widysanto A. GeneXpert Mycobacterium tuberculosis/ rifampicin assay for molecular epidemiology of rifampicin-resistant Mycobacterium tuberculosis in an urban setting of Banten province, Indonesia. *Int J Mycobacteriol* 2019;8:351-8.
23. Atashi S, Izadi B, Jalilian S. et al. Evaluation of GeneXpert MTB / RIF for determination of rifampicin resistance among new tuberculosis cases in west and northwest Iran. *New Microbes New Infect*. 2017;19:117-120. doi:10.1016/j.nmni.2017.07.002
24. Pang, Y. , Xia, H. , Zhang, Z., et al. Multicenter evaluation of genechip for detection of multidrug-resistant mycobacterium tuberculosis. *Journal of Clinical Microbiology*, 51(6), 1707-1713. 2013;51(6):1707-1713. doi:10.1128/JCM.03436-12
25. Yin J, Yuan J, Hu Y, Wei X. Association between directly observed therapy and treatment outcomes in multidrug-resistant tuberculosis: A systematic review and meta-analysis. *PLoS One*. 2016;11(3):1-14. doi:10.1371/journal.pone.0150511
26. Narges A , Leah J , Cecily M , et al. Adherence interventions and outcomes of tuberculosis treatment: A systematic review and meta-analysis of trials and observational studies. *PLoS Med*, 2018, 15(7):e1002595. doi:10.1371/journal.pmed.1002595
27. Baral SC, Aryal Y, Bhattrai R, et al. The importance of providing counselling and financial support to patients receiving treatment for multi-drug resistant TB: Mixed method qualitative and pilot intervention studies. *BMC Public Health.* 2014;14(1). doi:10.1186/1471-2458-14-46



For peer review only - http://bmjopen.bmj.com/site/about/guidelines.xhtml

28. Wang L, Li R, Xu C, et al. The Global Fund in China: Multidrug-resistant tuberculosis nationwide programmatic scale-up and challenges to transition to full country ownership. *PLoS One*. 2017;12(6):1-13. doi:10.1371/journal.pone.0177536

29. Subbaraman R, Nathavitharana RR, Mayer KH, et al. Constructing care cascades for active tuberculosis: A strategy for program monitoring and identifying gaps in quality of care. *PLoS Med*. 2019;16(2):1-18. doi:10.1371/journal.pmed.1002754

30. China National Bureau of Statistics. Accessed August, 2020 http://www.stats.gov.cn/tjsj/ndsj/2019/indexch.htm

31. Truzyan N, Crape B, Grigoryan R, et al. Increased risk for multidrug-resistant tuberculosis in migratory workers, Armenia. *Emerg Infect Dis*. 2015;21(3):474-476. doi:10.3201/eid2103.140474

32. Parmar MM, Sachdeva KS, Dewan PK, et al. Unacceptable treatment outcomes and associated factors among India's initial cohorts of multidrug-resistant tuberculosis (MDR-TB) patients under the revised national TB control programme (2007–2011): Evidence leading to policy enhancement. *PLoS One*. 2018;13(4):1-24. doi:10.1371/journal.pone.0193903

33. Lalor MK, Greig J, Allamuratova S, et al. Risk factors associated with default from multi- and extensively drug-resistant tuberculosis treatment, Uzbekistan: A retrospective cohort analysis. *PLoS One*. 2013;8(11). doi:10.1371/journal.pone.0078364

34. Li X, Yang Q, Feng B, et al. Tuberculosis infection in rural labor migrants in Shenzhen, China: Emerging challenge to tuberculosis control during urbanization. *Sci Rep*. 2017;7(1):1-8. doi:10.1038/s41598-017-04788-1

35. Zumla A, Abubakar I. Improving access to multi-drug resistant tuberculosis diagnostic and health services for refugees and migrants. *BMC Med*. 2018;16(1):7-10. doi:10.1186/s12916-018-1218-0

36. Wang W, Wang J, Zhao Q, et al. Contribution of rural-to-urban migration in the prevalence of drug resistant tuberculosis in China. Eur J Clin Microbiol Infect Dis. 2011;30(4):581-586. doi:10.1007/s10096-010-1125-6

37. Nellums LB, Rustage K, Hargreaves S, et a;. Multidrug-resistant tuberculosis treatment adherence in migrants: A systematic review and meta-analysis. *BMC Me*d. 2018;16(1):1-11. doi:10.1186/s12916-017-1001-7

38. Long Q, Li Y, Wang Y, et al. Barriers to accessing TB diagnosis for rural-to-urban migrants with chronic cough in Chongqing, China: A mixed methods study. *BMC Health Serv Res*. 2008;8:1-10. doi:10.1186/1472-6963-8-202

39. Wei X, Chen J, Chen P, et al. Barriers to TB care for rural-to-urban migrant TB patients in Shanghai: A qualitative study. *Trop Med Int Heal.* 2009;14(7):754-760. doi:10.1111/j.1365-3156.2009.02286.x

40. Wei X, Zou G, Yin J, et al. Providing financial incentives to rural-to-urban tuberculosis migrants in Shanghai: An intervention study. *Infect Dis Poverty.* 2012;1(1):1-8. doi:10.1186/2049-9957-1-9

**FIGURES AND LEGENDS**

Figure 1: Seven steps of the DRTB diagnosis and care cascade

Figure 2: Characteristics of diagnosed, registered and treated RR/MDR-TB patients, 2015-2018



For peer review only - http://bmjopen.bmj.com/site/about/guidelines.xhtml

Figure 3: Combination of DRTB policies implemented in each prefecture from 2015-2018

For peer review only



For peer review only - http://bmjopen.bmj.com/site/about/guidelines.xhtml

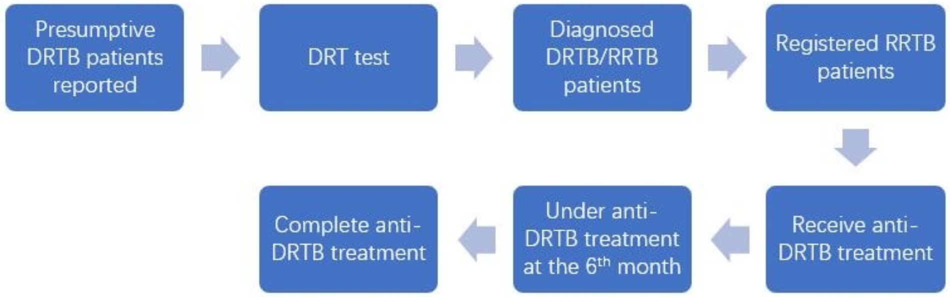


210x71mm (96 x 96 DPI)

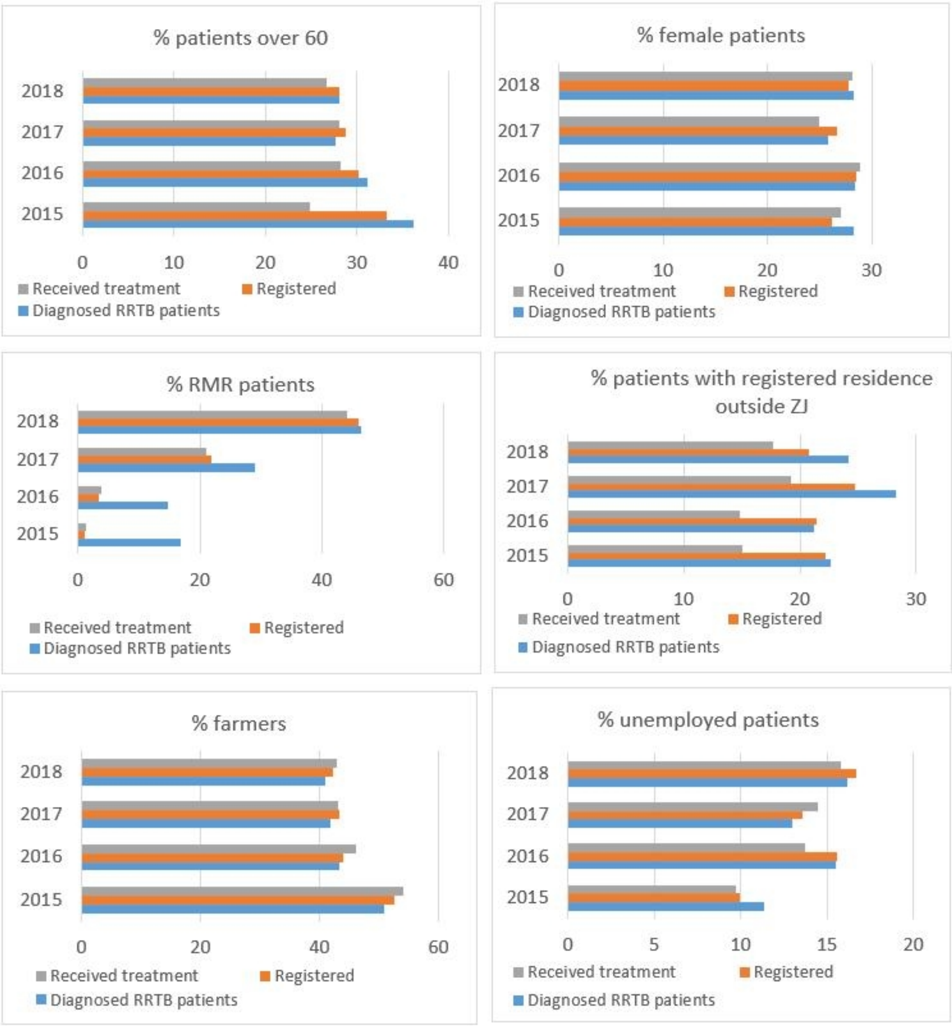


206x217mm (96 x 96 DPI)

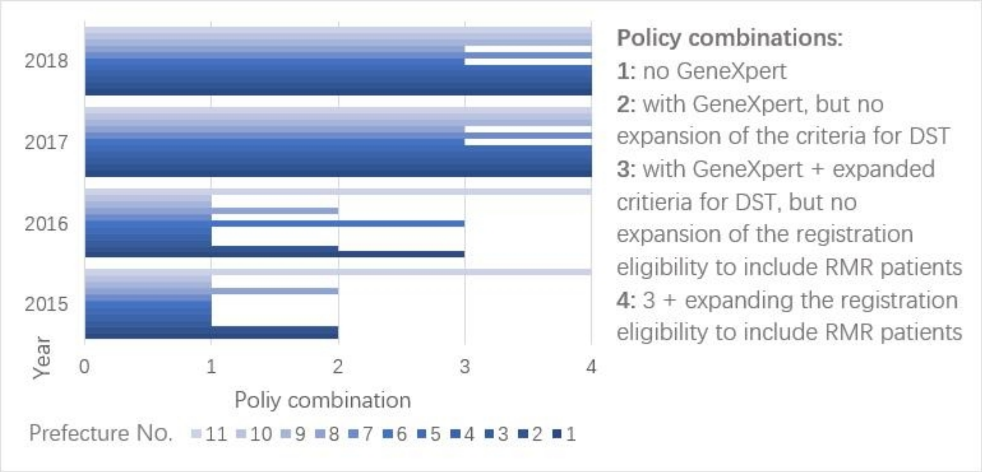


207x102mm (96 x 96 DPI)

**SUPPLEMENTAL MATERIALS**

Table 1: Year of policy change regarding the screening test and registration in the 11 prefectures

| Prefecture No. | Year of policy change | | |
|---|---|---|---|
| | screening range | test technology | registration policy |
| 1 | 2016 | 2015 | 2017 |
| 2 | 2017 | 2014 | 2014 |
| 3 | 2017 | 2017 | 2017 |
| 4 | 2017 | 2017 | 2017 |
| 5 | 2017 | 2017 | 2017 |
| 6 | 2009 | 2016 | 2019 |
| 7 | 2017 | 2017 | 2017 |
| 8 | 2017 | 2015 | 2019 |
| 9 | 2017 | 2017 | 2017 |
| 10 | 2017 | 2017 | 2015 |
| 11 | 2000 | 2012 | 2012 |

Table 2: Starting time and eligibility for health insurance and medical assistance policy coverage, 2015-2018

| Prefecture | Health insurance policy | | | Medical assistance | | |
|---|---|---|---|---|---|---|
| No. | starting time | insurance outside ZJ | RMR | starting time | registered residence outside ZJ | RMR |
| 1 | 2018 | N | Y | 2013 | N | N |
| 2 | 2013 | N | Y | 2013 | Y | Y since 2015 |
| 3 | 2016 | N | N | 2014 | Y | N |
| 4 | 2017 | N | Y | 2015 | Y | N |
| 5 | 2016 | N | N | 2015 | N | N |
| 6 | 2019 | N | N | 2012 | N | Y |
| 7 | 2014 | N | N | 2014 | Y | N |
| 8 | 2013 | N | N | 2015 | N | Y |
| 9 | 2014 | N | Y since 2017 | | No policy | |
| 10 | 2013 | N | Y | 2015 | N | Y |
| 11 | 2011 | N | Y | 2015 | Y | Y |

Table 3: The diagnostic test for presumptive DRTB patients from 2015-2018

| Year | No. of presumptive patients reported | % took fast DST | % took traditional DST | % took both tests | Patients with no DST records (%) | | |
|---|---|---|---|---|---|---|---|
| | | | | | total | not TB | unknown reason |
| 2015 | 9285 | 18.2 | 51.3 | 0.2 | 30.7 | 13.4 | 17.3 |
| 2016 | 10997 | 30.4 | 46.9 | 0.5 | 23.3 | 11.6 | 11.7 |
| 2017 | 21768 | 30.9 | 42.8 | 5.8 | 32.2 | 25.6 | 6.6 |
| 2018 | 23916 | 64.4 | 33.8 | 20.1 | 21.9 | 19.3 | 2.7 |

For peer review only

Table 4: Reasons for not completing anti-DRTB treatment according to the registration records

| Year | Total | Under treatment | | Dead | | lost-to-follow-up | | Other reasons | |
|---|---|---|---|---|---|---|---|---|---|
| | | No. | % | No. | % | No. | % | No. | % |
| 2015 | 68 | 11 | 16.2 | 17 | 25.0 | 13 | 19.1 | 27 | 39.7 |
| 2016 | 101 | 38 | 37.6 | 15 | 14.9 | 15 | 14.9 | 33 | 32.7 |

For peer review only

STROBE Statement—Checklist of items that should be included in reports of ***cross-sectional studies***

| | Item No | Recommendation | Page No |
|---|---|---|---|
| **Title and abstract** | 1 | (*a*) Indicate the study's design with a commonly used term in the title or the abstract | 1 |
| | | (*b*) Provide in the abstract an informative and balanced summary of what was done and what was found | 3 |
| **Introduction** | | | |
| Background/rationale | 2 | Explain the scientific background and rationale for the investigation being reported | 5 |
| Objectives | 3 | State specific objectives, including any prespecified hypotheses | 6 |
| **Methods** | | | |
| Study design | 4 | Present key elements of study design early in the paper | 7 |
| Setting | 5 | Describe the setting, locations, and relevant dates, including periods of recruitment, exposure, follow-up, and data collection | 7,8 |
| Participants | 6 | (*a*) Give the eligibility criteria, and the sources and methods of selection of participants | 8 |
| Variables | 7 | Clearly define all outcomes, exposures, predictors, potential confounders, and effect modifiers. Give diagnostic criteria, if applicable | NA |
| Data sources/ measurement | 8* | For each variable of interest, give sources of data and details of methods of assessment (measurement). Describe comparability of assessment methods if there is more than one group | *8* |
| Bias | 9 | Describe any efforts to address potential sources of bias | NA |
| Study size | 10 | Explain how the study size was arrived at | 8 |
| Quantitative variables | 11 | Explain how quantitative variables were handled in the analyses. If applicable, describe which groupings were chosen and why | 10 |
| Statistical methods | 12 | (*a*) Describe all statistical methods, including those used to control for confounding | 10 |
| | | (*b*) Describe any methods used to examine subgroups and interactions | 10 |
| | | (*c*) Explain how missing data were addressed | NA |
| | | (*d*) If applicable, describe analytical methods taking account of sampling strategy | NA |
| | | (*e*) Describe any sensitivity analyses | NA |
| **Results** | | | |
| Participants | 13* | (a) Report numbers of individuals at each stage of study—eg numbers potentially eligible, examined for eligibility, confirmed eligible, included in the study, completing follow-up, and analysed | 12,13 |
| | | (b) Give reasons for non-participation at each stage | NA |
| | | (c) Consider use of a flow diagram | NA |
| Descriptive data | 14* | (a) Give characteristics of study participants (eg demographic, clinical, social) and information on exposures and potential confounders | 14 |
| | | (b) Indicate number of participants with missing data for each variable of interest | NA |
| Outcome data | 15* | Report numbers of outcome events or summary measures | 12,13 |
| Main results | 16 | (*a*) Give unadjusted estimates and, if applicable, confounder-adjusted estimates and their precision (eg, 95% confidence interval). Make clear which confounders were adjusted for and why they were included | 15,16, 17,18 |

For peer review only

| | | | |
|---|---|---|---|
| | | (*b*) Report category boundaries when continuous variables were categorized | 10 |
| | | (*c*) If relevant, consider translating estimates of relative risk into absolute risk for a meaningful time period | NA |
| Other analyses | 17 | Report other analyses done—eg analyses of subgroups and interactions, and sensitivity analyses | NA |
| **Discussion** | | | |
| Key results | 18 | Summarise key results with reference to study objectives | 18 |
| Limitations | 19 | Discuss limitations of the study, taking into account sources of potential bias or imprecision. Discuss both direction and magnitude of any potential bias | 20 |
| Interpretation | 20 | Give a cautious overall interpretation of results considering objectives, limitations, multiplicity of analyses, results from similar studies, and other relevant evidence | 18,19 20 |
| Generalisability | 21 | Discuss the generalisability (external validity) of the study results | 20 |
| **Other information** | | | |
| Funding | 22 | Give the source of funding and the role of the funders for the present study and, if applicable, for the original study on which the present article is based | 21 |

*Give information separately for exposed and unexposed groups.

**Note:** An Explanation and Elaboration article discusses each checklist item and gives methodological background and published examples of transparent reporting. The STROBE checklist is best used in conjunction with this article (freely available on the Web sites of PLoS Medicine at http://www.plosmedicine.org/, Annals of Internal Medicine at http://www.annals.org/, and Epidemiology at http://www.epidem.com/). Information on the STROBE Initiative is available at www.strobe-statement.org.

For peer review only



For peer review only - http://bmjopen.bmj.com/site/about/guidelines.xhtml

# Policy changes and the screening, diagnosis and treatment of drug-resistant tuberculosis patients from 2015-2018 in Zhejiang Province, China: a retrospective cohort study


| | |
|---|---|
| Journal: | *BMJ Open* |
| Manuscript ID | bmjopen-2020-047023.R1 |
| Article Type: | Original research |
| Date Submitted by the Author: | 02-Mar-2021 |


| | |
|---|---|
| Complete List of Authors: | Jiang, Weixi; Duke Kunshan University, Global Health Research Center<br>Peng, Ying; Zhejiang Provincial Center for Disease Control and Prevention<br>Wang, Xiaomeng; Zhejiang Provincial Center for Disease Control and Prevention<br>Elbers, Chris; Vrije Universiteit Amsterdam, School of Business and Economics, Vrije Universiteit Amsterdam<br>Tang, Shenglan; Duke University, Duke Global Health Institute<br>Huang, Fei; Centers for Disease Control China, National Center for Tuberculosis Control and Prevention<br>Chen, Bin; Zhejiang Provincial Center for Disease Control and Prevention<br>Cobelens, Frank ; Amsterdam University Medical Centres, Department of Global Health and Amsterdam Institute for Global Health and Development |


| | |
|---|---|
| <b>Primary Subject Heading</b>: | Health policy |
| Secondary Subject Heading: | Public health |
| Keywords: | Tuberculosis < INFECTIOUS DISEASES, Health policy < HEALTH SERVICES ADMINISTRATION & MANAGEMENT, HEALTH SERVICES ADMINISTRATION & MANAGEMENT |
| | |


SCHOLARONE™
Manuscripts

*I, the Submitting Author has the right to grant and does grant on behalf of all authors of the Work (as defined in the below author licence), an exclusive licence and/or a non-exclusive licence for contributions from authors who are: i) UK Crown employees; ii) where BMJ has agreed a CC-BY licence shall apply, and/or iii) in accordance with the terms applicable for US Federal Government officers or employees acting as part of their official duties; on a worldwide, perpetual, irrevocable, royalty-free basis to BMJ Publishing Group Ltd ("BMJ") its licensees and where the relevant Journal is co-owned by BMJ to the co-owners of the Journal, to publish the Work in this journal and any other BMJ products and to exploit all rights, as set out in our licence.*

*The Submitting Author accepts and understands that any supply made under these terms is made by BMJ to the Submitting Author unless you are acting as an employee on behalf of your employer or a postgraduate student of an affiliated institution which is paying any applicable article publishing charge ("APC") for Open Access articles. Where the Submitting Author wishes to make the Work available on an Open Access basis (and intends to pay the relevant APC), the terms of reuse of such Open Access shall be governed by a Creative Commons licence – details of these licences and which Creative Commons licence will apply to this Work are set out in our licence referred to above.*

*Other than as permitted in any relevant BMJ Author's Self Archiving Policies, I confirm this Work has not been accepted for publication elsewhere, is not being considered for publication elsewhere and does not duplicate material already published. I confirm all authors consent to publication of this Work and authorise the granting of this licence.*

For peer review only

**Policy changes and the screening, diagnosis and treatment of drug-resistant tuberculosis patients from 2015-2018 in Zhejiang Province, China: a retrospective cohort study**

**Corresponding author:**
**Bin Chen**
Postal address: Zhejiang Provincial Center for Disease Control and Prevention, No. 3399 Binsheng Road, Binjiang District, Hangzhou, China
Email: bchen@cdc.zj.cn

**List of authors:**
**Weixi Jiang**
Global Health Research Center, Duke Kunshan University, Kunshan, Jiangsu, China
Email: weixi.jiang@dukekunshan.edu.cn

**Ying Peng**
Zhejiang Provincial Center for Disease Control and Prevention, Hangzhou, China
Email: ypeng@cdc.zj.cn

**Xiaomeng Wang**
Zhejiang Provincial Center for Disease Control and Prevention, Hangzhou, China
Email: xmwang@cdc.zj.cn

**Chris Elbers**
School of Business and Economics, Vrije Universiteit Amsterdam, Amsterdam, the Netherlands
Email: c.t.m.elbers@vu.nl

**Shenglan Tang**
Duke Global Health Institute, Duke University, Durham, NC, US
Email: shenglan.tang@duke.edu

**Fei Huang**
National Center for Tuberculosis Control and Prevention, China CDC, Beijing, China
Email: huangfei@chinacdc.cn

**Bin Chen**
Zhejiang Provincial Center for Disease Control and Prevention, Hangzhou, China
Email: bchen@cdc.zj.cn

**Frank Cobelens**
Department of Global Health and Amsterdam Institute for Global Health and Development, Amsterdam University Medical Centers, Amsterdam, the Netherlands



For peer review only - http://bmjopen.bmj.com/site/about/guidelines.xhtml

Email: f.cobelens@aighd.org

**Word count: 3823**

For peer review only



For peer review only - http://bmjopen.bmj.com/site/about/guidelines.xhtml

## ABSTRACT

**Objectives** To examine changes in the screening, diagnosis, treatment and management of drug-resistant tuberculosis (DRTB) patients, and investigate the impacts of DRTB related policies on patients of different demographic and socio-economic characteristics.

**Design** A retrospective cohort study using registry data, plus a survey on DRTB-related policies.

**Setting** All prefecture-level Centers for Disease Control in Zhejiang Province, China

**Main outcome measures** Alongside the care cascade we examined: 1) reported number of presumptive DRTB patients; 2) percentage of presumptive patients with drug susceptibility testing (DST) records; 3) percentage of DRTB/rifampicin-resistant (RRTB) patients registered; 4) percentage of RR/MDRTB patients that received anti-DRTB treatment; 5) percentage of RR/MDRTB patients cured/completed treatment among those treated. Multivariate logistic regressions were conducted to explore the impacts of DRTB policies after adjusting for other factors.

**Results** The number of reported presumptive DRTB patients and the percentage with DST records largely increased during 2015-2018, and the percentage of registered patients who received anti-DRTB treatment also increased from 59.0% to 86.5%. Patients under the policies of equipping GeneXpert plus expanded criteria for DST had a higher likelihood of being registered compared with no GeneXpert (adjusted odds ratio, aOR=2.57, 95% CI: 1.20, 5.51), while for treatment initiation the association was only when further expanding the registration criteria (aOR=2.38, 95% CI: 1.19, 4.79). Patients with registered residence inside Zhejiang were more likely to be registered (aOR=1.96, 95% CI 1.52, 2.52), treated (aOR=3.83, 95% CI 2.78, 5.28) and complete treatment (aOR=1.92, 95% CI 1.03, 3.59) compared with those outside.

**Conclusion** The policy changes on DST and registration have effectively improved DRTB case finding and care. Nevertheless, challenges remain in servicing vulnerable groups such as migrants and improving equity in the access to TB care. Future policies should provide comprehensive support for migrants to complete treatment at their current place of residence.


**Key words**: Tuberculosis, Health policy



For peer review only - http://bmjopen.bmj.com/site/about/guidelines.xhtml

**Article summary**

**Strengths and limitations of this study**

Strengths

- This study conducted a comprehensive and systematic cascade analysis on the healthcare pathway for drug-resistant tuberculosis (DRTB) patients: screening, diagnosis, treatment and management, and examined key factors associated with the case finding and healthcare process.
- This study evaluated the effectiveness of different combinations of DRTB related policies as they were implemented in all prefectures of one Chinese province, including new diagnostic technology promotion, improved patient management and financial support.
- This study also examined those patients left-behind during the policy changes from an equity perspective, with a focus on rifampin mono-resistant (RMR) patients and patients with registered residence (Hukou) outside Zhejiang.

Limitations

- The impacts of the financing policies could not be explored as almost all prefectures had some form of financing policies since 2015 and there were no individual-level data on whether the patient benefited from these financing policies.
- Other potentially important socio-economic factors like education and income were not available in the dataset used, limiting therefore the scope of the equity analysis to migrant workers.

For peer review only



For peer review only - http://bmjopen.bmj.com/site/about/guidelines.xhtml

## INTRODUCTION

Drug-resistant tuberculosis (DRTB) has become a global concern in recent years. It is estimated that globally in 2018, 3.4% of new TB patients and 18% of previously treated patients developed rifampicin-resistant forms of disease (RRTB) including multi-drug resistant TB (MDR-TB), in which there is additional resistance to isoniazid, and 7.1% and 21% respectively in China.[1] Currently China accounts for 14% of the RR/MDR-TB disease burden, and one recent projection suggested the incidence of RR/MDR-TB would triple without interventions to change current conditions.[2] The RR/MDR-TB epidemic has posed a great challenge to achieve the SDG target of ending TB in 2030 in China.[3]

The diagnosis and treatment of DRTB can be very costly in terms of both time and money,[4] and barriers to accessing DRTB diagnosis and care exist worldwide.[5] The diagnosis of DRTB takes 1-3 months using traditional technology, and the treatment of RR/MDR-TB lasts for up to two years. DRTB patients generally have lower socio-economic status, and the cost of treatment is so high that the current financing policies in the form of health insurance reimbursement and subsidized treatment are far from sufficient.[6-8] For China specifically, the public health insurance programs provide very limited coverage for outpatient services that are required for around 20 months for RR/MDR-TB patients. In addition, some second-line anti-TB drugs and auxiliary drugs are often not covered.[9-11] Previous studies in several countries including China have also revealed long treatment delay, high pre-diagnosis and pre-treatment attrition as and high loss-to-follow-up during the treatment course.[12-17] Moreover, the MDR-TB treatment success rate in China was less than 50%.[18]

These formidable barriers for DRTB patients to accessing and adhering to standard treatment call



For peer review only - http://bmjopen.bmj.com/site/about/guidelines.xhtml

for strong supporting policies for patients to receive and complete treatment.[19] Previous studies have validated the utility of rapid drug susceptibility testing (DST) technologies such as Genechip and GeneXpert in the screening for DRTB, and shown that these technologies could improve DRTB case finding, shorten treatment delays and decrease pre-diagnosis attrition.[13, 20–24] As for treatment adherence, one comprehensive program in China that provided universal health coverage to MDR-TB patients was shown to improve access to and affordability of diagnosis and quality treatment of MDRTB.[5] Studies on other intervention strategies showed that directly observed therapy (DOT) or other reminding approaches through digital technologies could improve the treatment adherence and outcomes of TB/MDRTB.[25, 26] Patient counseling alone or combined with financial support can also increase the cure rates among MDRTB patients.[27]

In China some provinces, along with or after the roll-out of international donor-funded projects, have implemented policies to improve DRTB control, including allocating special funds to equip DRTB-designated hospitals with DST facilities and reagents, improving health insurance benefit packages and providing subsidies to patients.[11,28] However, there is limited evidence on the impact of implementing these policies on DRTB case finding and care thereafter. The equity issues underlying the case finding and care procedures for DRTB patients are also understudied, especially considering that the eligibility for policies issued in a certain region is often linked with patients' registered residence, work and health insurance status.

This study aims to examine changes in the programmatic performance with regard to screening, diagnosis, treatment and management of DRTB patients in Zhejiang from 2015 to 2018 through a cascade analysis approach.[29] In addition, as the policies and guidelines on this whole procedure of DRTB care changed during this period, this study systematically summarizes these changes and



For peer review only - http://bmjopen.bmj.com/site/about/guidelines.xhtml

investigates how these policy changes have influenced case finding and treatment of DRTB patients. We also explore whether these policy changes have equally influenced patients of different demographic and socio-economic characteristics.

## METHODS

### Study settings

Zhejiang is a province located in the eastern area with its GDP ranking 4th in China,[30] and has a growing migrant population in recent years. Under the current TB control model in Zhejiang, the prefecture-level designated hospital, usually one in each prefecture, is responsible for the diagnosis and treatment of DRTB patients, while the Center for Disease Control (CDC) and primary health care facilities conduct patient management. Patients who are clinically suspected to have DRTB are to be referred for DST and should be reported in the Tuberculosis Information Management System (TBIMS) as presumptive DRTB patients. Their sputum samples are to be sent to the prefecture-level designated hospital for DST. If diagnosed with DRTB, these patients should be registered in the TBIMS database for diagnosed patients as such. The criteria regarding what types of DRTB should be registered have being changing over time.

### Study Design

This study included a questionnaire survey on the DRTB policies/programs among the 11 prefecture-level CDCs and a quantitative analysis of the TBIMS records of presumptive and diagnosed DRTB patients.

Questionnaire survey on DRTB policies/programs



For peer review only - http://bmjopen.bmj.com/site/about/guidelines.xhtml

A questionnaire on the DRTB related policies was distributed to the CDC of all prefectures in Zhejiang in collaboration with Zhejiang provincial CDC. After preliminary consultation with the provincial CDC, the questionnaire was designed to include policies in four areas: 1) eligibility of presumptive DRTB patients for DST; 2) eligibility for registering DRTB patients based on the type of drug-resistance (including rifampin mono-resistance (RMR), multi-drug resistance(MDR), extensive drug-resistance (XDR) and mono-resistance to other types of drugs); 3) DST technology and payment for DST and 4) financing policies, including both health insurance benefit packages and government subsidies for supporting the DRTB treatment. Detailed questions on the eligibility for financial support regarding the types of DRTB, the registered permanent residence and the region of health insurance enrollment were also included in the questionnaire (see supplementary materials). If the policies had changed at any time after 2015, we collected information on the policy details before and after the change as well as the year of change.

TBIMS records

De-identified TBIMS records of presumptive and diagnosed DRTB patients from 2015-2018 in Zhejiang were retrieved from the TB Prevention and Control Center of China CDC. The dataset of presumptive DRTB patients included demographic information (prefecture of registration, age, sex, ethnicity, occupation and registered residence), drug-resistance test profile (the date of sending the sample, conducting the test and reporting the result, the type of test and the test result), as well as a unique registration number if registered as a DRTB patient in the diagnosed patient dataset. Only those DRTB patients recorded in the diagnosed patient dataset have a traceable treatment history and are managed under a specialized guideline for DRTB patients. The dataset of diagnosed DRTB patients contained the same demographic information and diagnostic data as the dataset of



For peer review only - http://bmjopen.bmj.com/site/about/guidelines.xhtml

presumptive patients, plus the treatment information including the starting date of anti-DRTB treatment including both second-line (RR/MDR-TB) and adapted first-line (monoresistance to isoniazid) treatment, TB treatment history, current state of treatment (under treatment or ended), and the ending date of treatment as well as the reason of ending the treatment (cured/treatment completed/death/lost-to-follow-up/others) if ended. In TBIMS the treatment status referred to whether the patient received treatment in Zhejiang. If patients were registered but returned to their hometown for treatment, it could be shown as "treatment refused".

**Patient and Public Involvement Statement**

Patients or the public were not involved in the design, or conduct, or reporting, or dissemination plans of our research.

**Data analysis**

Using datasets of the presumptive and registered diagnosed patients, a seven-step cascade of diagnosis and care was constructed for each year from 2015 to 2018 : 1) reported presumptive DRTB patients; 2) presumptive patients who had DST; 3) diagnosed DRTB, and RR/MDRTB (including RMR, MDR and XDR) patients; 4) registered RR/MDRTB patients; 5) RR/MDRTB patients that ever received anti-DRTB treatment; 6) RR/MDRTB patients that were under treatment 6 months after treatment initiated; 7) RR/MDRTB patients that had been cured or completed treatment, as shown in Figure 1. Diagnosed DRTB patients were defined as patients with a DST result showing resistance to at least one type of first-line anti-TB drug. As in all but one prefectures only RR/MDRTB patients were required to be registered and thus had available data on treatment history



For peer review only - http://bmjopen.bmj.com/site/about/guidelines.xhtml

before 2019, we only analyzed RR/MDRTB patients for step 4-7. Descriptive analyses were conducted to explore the reasons for attrition at each step based on the relevant records in the datasets. Patients' ethnicity was categorized as Han and other minority groups. Frequencies of patient characteristics were also calculated across patient groups at each step of the cascade to examine potential factors associated with the attrition. For step 3-5 the frequencies of some patient characteristics of interests, including age, sex, drug-resistant type, occupation and registered residence were calculated separately for each year to examine the trends from 2015 to 2018.

Mixed-effect two-level logistic regressions, with fixed slope and random intercept specification were conducted to explore factors associated with the likelihood of being registered for RRTB patients, the likelihood of receiving anti-DRTB treatment if registered, and the likelihood of getting cured or completing treatment (the latter only for those who initiated treatment before 2017 because of the 2-year treatment duration). This model showed better fit to the data than the fixed-effect model based on the likelihood-ratio test. The DRTB related policies were summarized along the four areas in the questionnaires. Besides patient-level variables, the policies implemented in each prefecture and each year, categorized according to the different combinations of policies in the four areas, were included in the model. Per-capita GDP of the prefecture was divided into three groups, as the numbers assembled in three intervals: RMB 55,000-70,000, 80,000-100,000, and above 120,000, and also included in the model as a categoric variable. Standard errors were estimated accounting for clustering, considering the intra-prefecture correlations.

## RESULTS

### DRTB policy change from 2015-2018 in Zhejiang



For peer review only - http://bmjopen.bmj.com/site/about/guidelines.xhtml

Results from the questionnaire survey on DRTB policies showed that the major change in the eligibility of patients for DST was the expansion from high-risk patients (including smear positive relapse, treatment failure, positive sputum bacteriology after two months' treatment), to all bacteriologically positive TB patients. As for the testing technology, the major change was the introduction of the rapid DST assay GeneXpert MTB/RIF that combines testing for *Mycobacterium tuberculosis* with screening for rifampicin-resistance with a same-day result, while the costs for the test were all covered, or mostly covered by the government. The criteria for DRTB patient registration (implying eligibility for specialized DRTB patient management) also expanded from MDR/XDR-TB to RR-TB, and one prefecture even expanded to any DRTB before 2018. In most prefectures these changes in DST eligibility and equipment as well as the registration criteria happened in 2017, while almost all others had adopted the new polices and introduced GeneXpert earlier (supplemental table 1).

The financing policies to support DRTB treatment in Zhejiang consisted of both health insurance and medical assistance policies, and in most prefectures these policies had been implemented in 2015. These financing policies aimed to improve the benefit package for DRTB patients through a three-layer coverage system: 1) increasing the reimbursement rate of outpatient treatment to 70%-90%, the same level as for inpatient treatment; 2) the out-of-pocket (OOP) expenditure exceeding a certain amount, typically RMB 20,000-30,000, could be further reimbursed at a rate of 55%-85% through a supplemental health insurance program for critical illnesses; 3) the OOP expenditure after health insurance reimbursement could be covered by the medical assistance, with a payment limit varying from 11,000 RMB to over 60,000 RMB across prefectures. As for the eligibility for the insurance policies, those with public health insurance enrolled outside Zhejiang, usually migrants



For peer review only - http://bmjopen.bmj.com/site/about/guidelines.xhtml

without formal jobs, were excluded. In 5 out of the 11 prefectures RMR patients were still not eligible for the expanded public health insurance benefit package up to 2019. For the medical assistance, in 6 out of the 11 prefectures only patients with registered residence ("Hukou") in Zhejiang were eligible, and for the other 4 prefectures with assistance policies the eligibility had been expanded to all residents in this prefecture. Up to 2018, RMR patients in 5 prefectures were still excluded from the medical assistance (supplemental table 2).

**Cascade analysis of DRTB care from 2015-2018**

Table 1 shows the screening and diagnosis of DRTB patients from 2015-2018. In general, the results suggested increased capacity to find DRTB patients. The number of reported presumptive DRTB patients increased from 9285 to 23916, with the largest increase from 2016 to 2017 coinciding with the change in screening policy in most prefectures. The percentage of patients with test results also increased from 69.3% to 78.1%. Along with the increase in the volume of DST there was an increase in the number of diagnosed DRTB patients except for 2018, and a decrease in the percentage of patients diagnosed among those tested. RR/MDR-TB patients accounted for around 40%-50% of the total DRTB patients.

Further analysis on the types of diagnostic tests patients received revealed an increasing trend in the percentage of patients taking rapid DST as well as the percentage of patients tested with both conventional culture-based and rapid DST. The percentage of presumptive patients without DST profile for unknown reasons as recorded in the TBIMS dataset, which indicates pre-diagnosis attrition, dropped dramatically from 17.3% to 2.7% (supplemental table 3).



For peer review only - http://bmjopen.bmj.com/site/about/guidelines.xhtml

Table 1: Diagnostic cascade starting from presumptive DRTB patients.

| Year | No. of presumptive DRTB patients | Patients with test records | | Diagnosed DRTB patients | | Diagnosed RR/MDR-TB patients | |
|---|---|---|---|---|---|---|---|
| | | No. | % | No. | % | No. | % |
| 2015 | 9285 | 6434 | 69.3 | 1031 | 16.0 | 503 | 48.8 |
| 2016 | 10997 | 8438 | 76.7 | 1258 | 14.9 | 529 | 42.1 |
| 2017 | 21768 | 14764 | 67.8 | 1729 | 11.7 | 716 | 41.4 |
| 2018 | 23916 | 18670 | 78.1 | 1580 | 8.5 | 663 | 42.0 |

Table 2 shows the registration and treatment for diagnosed RRTB patients from 2015-2018. While the actual number of registered RRTB patients increased, the percentage of registered RRTB patients dropped in 2017, and this percentage increased again to 84.6%. The percentage of registered patients that received anti-DRTB treatment increased from 59.0% to 86.5%, and the percentage of those treated who received at least 6 month's treatment remained above 90%. Around 70% of patients diagnosed in 2015 and 2016 completed treatment. Duration of treatment could be longer than 2 years, as 38 out of the 283 patients starting treatment in 2016 were still shown as under treatment at the time we retrieved the data (see supplemental table 4).

Table 2: Registration and treatment cascade for diagnosed RRTB patients.

| Year | Diagnosed RR/MDR-TB patients | Registered | | Received anti-DRTB treatment | | Under treatment 6 months after treatment initiated | | Cured or completed treatment | |
|---|---|---|---|---|---|---|---|---|---|
| | | No. | % | No. | % | No. | % | No. | % |
| 2015 | 503 | 383 | 76.1 | 226 | 59.0 | 215 | 95.1 | 158 | 73.5 |
| 2016 | 529 | 410 | 77.5 | 283 | 69.0 | 263 | 92.9 | 182 | 69.2 |
| 2017 | 716 | 502 | 70.1 | 360 | 71.7 | 334 | 92.8 | / | / |
| 2018 | 663 | 561 | 84.6 | 485 | 86.5 | 462 | 95.3 | / | / |

**Factors associated with the registration and treatment of DRTB patients**

Table 3 shows the characteristics of patients across each step of the care cascade. Around 70% of



For peer review only - http://bmjopen.bmj.com/site/about/guidelines.xhtml

the presumptive and diagnosed patients were male, and around 98% of them were Han People. Only around 40% of the patients had formal jobs other than farming or unemployment. While under the assumption of perfect equity we would expect that the percentage of patients with different characteristics remained the same from the diagnosed to the treated group, it decreased from 30.3% to 27.0% for older patients, 28.1% to 22.5% for RMR patients, and from 24.4 to 17.0% for patients with registered residence outside Zhejiang, i.e., migrants. An increase was observed regarding the percentage of patients ever taking rapid DST.

Table 3: Patient characteristics across each step of the care cascade

| | Presumptive DRTB patients | Diagnosed DRTB patients | Diagnosed RR/MDR-TB patients | Registered RR/MDR-TB patients | RR/MDR-TB patients recevied treatment |
|---|---|---|---|---|---|
| N | 65966 | 5598 | 2411 | 1859 | 1357 |
| Age (%) | | | | | |
| >=60 | 41.3 | 35.9 | 30.3 | 29.8 | 27.0 |
| Gender (%) | | | | | |
| Male | 70.6 | 73.6 | 72.5 | 72.7 | 72.7 |
| Ethnicity | | | | | |
| Han (%) | 97.7 | 98.0 | 97.9 | 98.0 | 98.8 |
| Patient type (%) | | | | | |
| new patient | 70.2 | 65.2 | 51.8 | 51.4 | 48.8 |
| Drug-resistanct type (%) | | | | | |
| RMR | / | 12.1 | 28.1 | 20.8 | 22.5 |
| MDR | / | 30.4 | 70.6 | 78.0 | 76.1 |
| XDR | / | 0.5 | 1.2 | 1.2 | 1.4 |
| Non-rifampicin resistant | / | 56.9 | / | / | / |
| Registered residence (%) | | | | | |
| outside Zhejiang | 23.4 | 25.2 | 24.4 | 22.3 | 17.0 |
| Job category (%) | | | | | |
| farmers | 49.0 | 48.0 | 43.8 | 45.0 | 45.5 |
| unemployed | 12.4 | 13.0 | 14.1 | 14.2 | 14.0 |
| other | 38.6 | 39.0 | 42.1 | 40.8 | 40.5 |
| Test type (%) | | | | | |
| fast test | / | 38.6 | 46.5 | 46.1 | 49.2 |



For peer review only - http://bmjopen.bmj.com/site/about/guidelines.xhtml

Figure 2 shows the changes in the characteristics of diagnosed, registered and treated RR/MDR-TB patients from 2015-2018. The percentage of diagnosed RMR patients that received adequate treatment was low in 2015 and 2016, but increased dramatically from 2017, coinciding with the policy change. The gaps between the proportion of older patients in the diagnosed and the treated group also narrowed. Nevertheless, over the four years there remained a 7-10 percentage decrease in the proportion of patients with registered residence outside Zhejiang from the diagnosed to the treated patient group, indicating that migrants were still more likely to drop out after diagnosis compared with the local residents. Gender and job category were not obviously associated with the pre-treatment attrition in this univariate analysis.

To systematically investigate the impact of policies on DRTB case finding and care, we divided the combination of policies in different areas of DRTB implemented in each prefecture for each year during 2015-2018 into four categories, considering both policy implications and the number of cases in each category. As shown in Figure 3, since 2017 all prefectures had equipped GeneXpert and expanded the eligibility criteria for DST as well as registration, except for two prefectures which still did not register RMR patients.

Table 4 shows the results of mixed-effect two-level logistic regression of the factors associated with registration among diagnosed RRTB patients, as well as with receiving anti-DRTB treatment among those registered. After adjusting for other factors, patients under the policy of providing GeneXpert together with expanding eligibility for DST (category 3, adjusted odds ratio (aOR) =2.57, 95% CI: 1.20, 5.51) had a significantly higher likelihood of being registered compared to patients not provided with GeneXpert, while this association was not significant for providing GeneXpert testing without expanding eligibility (category 2). A positive association with receiving anti-DRTB



For peer review only - http://bmjopen.bmj.com/site/about/guidelines.xhtml

treatment was significant only for the policy combination of providing GeneXpert and expanding eligibility for both DST and registration (category 4, aOR=2.38, 95%CI: 1.19, 4.79). Not surprisingly, MDR/XDR-TB patients were much more likely to be registered compared with RMR patients due to the registration policy in earlier time periods. In addition, patients with registered residence inside Zhejiang were more likely to be registered (aOR=1.96, 95% CI 1.52, 2.52) or treated (aOR=3.83, 95% CI 2.78, 5.28). Older age was associated with lower likelihood both of being registered (aOR=0.69,95% CI 0.53, 0.90) and of receiving anti-DRTB treatment (aOR=0.40, 95% CI 0.30, 0.52).

Tables 4: Factors associated with registration for diagnosed RRTB patients, and with receiving anti-DRTB treatment for registered RRTB patients.

| | Registration (n=2367) | | | | Receiving treatment (n=1824) | | | |
|---|---|---|---|---|---|---|---|---|
| | OR | P>z | 95% CI | | OR | P>z | 95%CI | |
| Age | | | | | | | | |
| >=60 | 0.69 | 0.006 | 0.53 | 0.90 | 0.40 | 0.000 | 0.30 | 0.52 |
| Gender | | | | | | | | |
| male | 1.08 | 0.554 | 0.85 | 1.37 | 0.87 | 0.425 | 0.62 | 1.22 |
| DR type | | | | | | | | |
| MDR/XDR-TB | 5.93 | 0.000 | 3.10 | 11.36 | 0.91 | 0.789 | 0.45 | 1.84 |
| Patient type | | | | | | | | |
| new patient | 1.22 | 0.146 | 0.93 | 1.59 | 0.52 | 0.000 | 0.37 | 0.72 |
| Treatment history | NA | | | | | | | |
| no treatment | | | | | ref. | | | |
| 1st-line drug only | | | | | 0.58 | 0.044 | 0.34 | 0.99 |
| 2nd-line drug used | | | | | 1.11 | 0.748 | 0.58 | 2.12 |
| Policy category | | | | | | | | |
| 1 | ref. | | | | ref. | | | |
| 2 | 1.18 | 0.626 | 0.61 | 2.29 | 0.87 | 0.776 | 0.34 | 2.25 |
| 3 | 2.57 | 0.015 | 1.20 | 5.51 | 1.44 | 0.257 | 0.77 | 2.68 |
| 4 | 2.08 | 0.054 | 0.99 | 4.37 | 2.38 | 0.015 | 1.19 | 4.79 |
| Registered residence | | | | | | | | |
| in Zhejiang | 1.96 | 0.000 | 1.52 | 2.52 | 3.83 | 0.000 | 2.78 | 5.28 |
| Job category | | | | | | | | |
| farmers | ref. | | | | ref. | | | |
| unemployed | 1.21 | 0.017 | 1.04 | 1.42 | 0.90 | 0.651 | 0.58 | 1.40 |



For peer review only - http://bmjopen.bmj.com/site/about/guidelines.xhtml

For peer review only

| | | | | | | | | |
|---|---|---|---|---|---|---|---|---|
| other jobs | 0.92 | 0.281 | 0.80 | 1.07 | 0.94 | 0.784 | 0.60 | 1.47 |
| Per capita GDP | | | | | | | | |
| highest group | ref. | | | | ref. | | | |
| middle group | 1.72 | 0.122 | 0.87 | 3.42 | 2.20 | 0.025 | 1.10 | 4.38 |
| lowest group | 1.70 | 0.099 | 0.90 | 3.20 | 1.15 | 0.643 | 0.63 | 2.12 |
| _cons | 0.32 | 0.002 | 0.16 | 0.66 | 1.85 | 0.207 | 0.71 | 4.78 |
| prefecture var(_cons) | 0.18 | | 0.09 | 0.39 | 0.18 | | 0.03 | 0.92 |

Tables 5 shows the results of the mixed-effect two-level logistic regression analysis of factors associated with the likelihood of completing treatment for patients who initiated treatment before 2017. All prefectures had some kind of financing policies in 2015 and 2016, and most prefectures had not yet expanded the eligibility of patients for DST or registration. We therefore used the individual level data on utilization of rapid DST to assess the impact of rapid testing on treatment completion. After adjusting for other factors, older patients were less likely to complete treatment (aOR=0.24, P<0.001), while registered residence inside Zhejiang was associated with higher probability of completing treatment (aOR=1.92, P=0.04). These two factors had significant impacts of the same direction on the registration, treatment initiation and treatment completion of RR/MDRTB patients. Utilization of rapid DST was not associated with treatment completion in this study.

Table 5: Factors associated with the likelihood of completing treatment for patients that initiated treatment before 2017

| | OR | P-value | 95% Conf. | Interval |
|---|---|---|---|---|
| Age | | | | |
| >=60 | 0.24 | <0.001 | 0.14 | 0.42 |
| Gender | | | | |
| male | 0.79 | 0.442 | 0.44 | 1.44 |
| Drug-resistance type | | | | |
| MDR/XDR-TB | 0.50 | 0.086 | 0.23 | 1.10 |
| Patient type | | | | |
| new patient | 1.06 | 0.820 | 0.64 | 1.77 |
| TB treatment history | | | | |



For peer review only - http://bmjopen.bmj.com/site/about/guidelines.xhtml

| | | | | |
|---|---|---|---|---|
| no treatment history | ref. | | | |
| first-line drug only | 0.74 | 0.355 | 0.39 | 1.40 |
| second-line drug used | 1.07 | 0.847 | 0.56 | 2.02 |
| Test type | | | | |
| fast test | 0.70 | 0.096 | 0.46 | 1.06 |
| Registered residence | | | | |
| in Zhejiang | 1.92 | 0.040 | 1.03 | 3.59 |
| Job category | | | | |
| farmers | ref. | | | |
| unemployed | 0.84 | 0.635 | 0.42 | 1.71 |
| other jobs | 1.44 | 0.278 | 0.74 | 2.79 |
| Per capita GDP | | | | |
| highest group | ref. | | | |
| middle group | 0.94 | 0.939 | 0.20 | 4.39 |
| lowest group | 1.07 | 0.921 | 0.30 | 3.74 |
| Year of sending sample | | | | |
| 2015 | ref. | | | |
| 2016 | 1.23 | 0.164 | 0.92 | 1.64 |
| _cons | 5.58 | 0.001 | 2.01 | 15.50 |
| prefecture var(_cons) | 0.21 | | 0.04 | 1.10 |

## DISCUSSION

Results from this study clearly revealed the positive impacts of the combined policy changes regarding DST of presumptive DRTB patients and registration for proper management of diagnosed patients. However, inequity challenges remain in terms of servicing vulnerable groups, e.g. migrant workers and the older population, in the registration, treatment and management of DRTB.

### Effectiveness and challenges of the policy interventions

The expansion of the eligibility criteria of presumptive DRTB patients referred for DST, together with the increased funding support to equip the facilities and guarantee the supply of reagents for rapid DST like GeneXpert, have greatly improved the capacity for DRTB case finding. The significant effects of the combined policies compared to providing GeneXpert alone indicates that



For peer review only - http://bmjopen.bmj.com/site/about/guidelines.xhtml

in order to effectively improve case finding we need to not just introduce new technologies, but also support and expand their use. The inclusion of RMR in the registration and management of DRTB patients closed the management gap between RMR and MDR/XDR-TB patients, and also improved the treatment rate for RR/MDR-TB.

It is obvious that these reforms in the DRTB policies and regulations have effectively changed the practices in DRTB control. Nevertheless, no policies aiming at migrants were issued between 2015 to 2018, and during these four years the degree of inequity between local and migrant patients did not seem to be mitigated either. The improved insurance benefit package was only available for those with public health insurance enrollment in Zhejiang, and in many prefectures receiving medical assistance still required local registered residence during 2015-2018. In addition, older people were less likely to be registered or receive treatment. This may be because they were more likely to give up treatment due to the high cost and long course of the standard treatment, and health workers may not register them once they refused to provide information necessary for registration. All these findings were consistent with previous studies that age and migration for work, as well as health system factors such as lack of clear eligibility criteria for DST and limited capacity to provide DST were associated with attrition at different stages of the cascade.[14, 16, 31–33] Nevertheless, no impacts were observed of other factors, such as association between treatment experience and treatment outcome, and data on other socio-economic factors such as financial difficulties were not available in this study.

**Equity challenges regarding migrant populations**

The growing number of migrants in China and other parts of the world are posing a challenge to TB



For peer review only - http://bmjopen.bmj.com/site/about/guidelines.xhtml

control.[31, 34–36] Although one systematic review found no significant differences in treatment adherence between migrant and long-term resident MDRTB patients,[37] several studies identified barriers for migrants to accessing TB diagnosis and care mainly in terms of knowledge gaps and financial difficulty,[38, 39] and these barriers would likely to be more substantial for migrant MDRTB patients. In our study, only around 40% of the DRTB patients had a formal job other than farmers or self-employment. In China people with a formal job will be compulsively enrolled in the urban employee basic medical insurance at the place of work as required by law. However, migrants without a formal job often choose to participate in the health insurance for residents in their hometown which requires lower premium compared to the more developed region they migrated to, and thus cannot benefit from the reimbursement policy in their working place. Besides, they often have to pay the full cost of treatment first before they get reimbursed when they return home. Therefore, it is expected that many of these DRTB patients would choose to go back to their hometown for treatment, or even refuse treatment. The need for migrant DRTB patients to travel long-distances and the substandard or even absent treatment received would increase the risk of disease transmission and treatment failure. A study in Shanghai, a well-developed city in eastern China, showed that financial incentives were effective for migrant TB patients to complete treatment.[40] In terms of these findings, policies aimed at encouraging migrant DRTB patients to be treated and managed at their current place of residence need to be developed, and this is particularly urgent for regions with a large migrant population.

**Limitations**

One major limitation of our study is that almost all prefectures had some form of financing policy since 2015 and there were no individual-level data on whether the patient benefited from these



For peer review only - http://bmjopen.bmj.com/site/about/guidelines.xhtml

financing policies. Therefore, in this study the impacts of these financing policies could not be explored. Furthermore, other potentially important socio-economic factors like education and income were not available in our dataset, limiting the scope of the equity analysis. Nevertheless, these limitations would not influence our conclusions that changes in the screening and registration policies of DRTB patients have largely increased the case finding and management capacity of DRTB patients, while during the period 2015-2018 differences between the local and migrant patients in the registration, treatment initiation and treatment outcome remained. We did not consider population changes either as the resident population only increased 5% from 2015 to 2018 in Zhejiang.[41] Future research should collect more individual-level data on the implementation of the health insurance and medical assistance policies to investigate the impacts of these demand-side policies on DRTB treatment more directly, and explore what kind of financing policies provided for migrants could increase their likelihood of initiating and completing anti-DRTB treatment.

**DECALRATIONS**

**Ethical approval**

This study is under the overall evaluation study of China-Gates TB Project Phase III and used data collected from this project. The implementation of this project has received the ethical approval from China CDC (No.201626). The protocol of the overall study design and data collection tools was reviewed and approved by the Institutional Review Board of Duke University (IRB Approval code: 2017-0768). This paper does not use data involving human participants.

**Competing interests**

All authors declare no competing interests.

**Funding**

The work was supported by the Bill and Melinda Gates Foundation grant number [OPP1149395].

**Contributorship statement**

The study was designed by Weixi Jiang, Shenglan Tang, Chris Elbers and Frank Cobelens. Fei Huang, Ying Peng, Xiaomeng Wang, Bin Chen and Weixi Jiang coordinated and conducted data collection. Weixi Jiang conducted literature review and wrote the manuscript as the first author. Shenglan Tang, Chris Elbers, Bin Chen and Frank Cobelens provided suggestions on data analysis



For peer review only - http://bmjopen.bmj.com/site/about/guidelines.xhtml

framework and data interpretation, and also revised the manuscript. All authors reviewed the draft manuscript and provided comments on the finalization of the manuscript. All authors have read and approved the manuscript in its current state.

**Data sharing statement**

Data may be obtained from a third party and are not publicly available. The data from the TBIMS used in this study are owned by China CDC. The data could only be accessed after obtaining permission from China CDC.

**Acknowledgements**

This paper is part of the outputs emanating from the program entitled "China National Health and Family Planning Commission and the Gates Foundation TB Project (Phase III)"-a collaboration between the Government of China and the Melinda and Bill Gates Foundation, and implemented by the China Center of Disease Control and Prevention (CDC). The authors of the paper also gratefully acknowledge the officers in Zhejiang Provincial CDC and the 11 prefectural CDCs for helping with collecting DRTB related policies in Zhejiang.

**REFERENCES**

1. World Health Organization. Global Tuberculosis Report 2019: P58
2. Li BY, Shi WP, Zhou CM, et al. Rising challenge of multidrug-resistant tuberculosis in China: A predictive study using Markov modeling. *Infect Dis Poverty.* 2020;9(1):1-8. doi:10.1186/s40249-020-00682-7
3. World Health Organization. accessed August, 2020 https://www.who.int/sdg/targets/en/
4. Fitzpatrick C, Floyd K. A Systematic Review of the Cost and Cost Effectiveness of Treatment for Multidrug-Resistant Tuberculosis. *Pharmacoeconomics*. 2012;30(1):63-80. doi:10.2165/11598640-000000000-00000
5. Li R, Ruan Y, Sun Q, et al. Effect of a comprehensive programme to provide universal access to care for sputum-smear-positive multidrug-resistant tuberculosis in China: A before-and-after study. *Lancet Glob Heal.* 2015;3(4):e217-e228. doi:10.1016/S2214-109X(15)70021-5
6. Long Q, Qu Y, Lucas H. Drug-resistant tuberculosis control in China: progress and challenges. *Infect Dis Poverty*. 2016;5(1):9. doi:10.1186/s40249-016-0103-3
7. Hutchison C, Khan MS, Yoong J, Lin X, Coker RJ. Financial barriers and coping strategies: a qualitative study of accessing multidrug-resistant tuberculosis and tuberculosis care in Yunnan, China. *BMC Public Health*. 2017;17(1):1-11. doi:10.1186/s12889-017-4089-y
8. Wingfield T, Boccia D, Tovar M, et al. Defining Catastrophic Costs and Comparing Their Importance for Adverse Tuberculosis Outcome with Multi-Drug Resistance: A Prospective Cohort Study, Peru. *PLoS Med*. 2014;11(7). doi:10.1371/journal.pmed.1001675
9. Pan H-Q, Bele S, Feng Y, et al. Analysis of the economic burden of diagnosis and treatment of tuberculosis patients in rural China. *Int J Tuberc Lung Dis.* 2013;17(12):1575-1580. doi:10.5588/ijtld.13.0144
10. Wang Y, McNeil EB, Huang Z, et al. Household financial burden among multidrug-resistant tuberculosis patients in Guizhou province, China: A cross-sectional study. *Med.* 2020;99(28):e21023. doi:10.1097/MD.0000000000021023



For peer review only - http://bmjopen.bmj.com/site/about/guidelines.xhtml

11. Tang S, Wang L, Wang H, Chin DP. Access to and affordability of healthcare for TB patients in China: Issues and challenges. *Infect Dis Poverty.* 2016;5(1):1-5. doi:10.1186/s40249-016-0096-y
12. Hemant Deepak Shewade, Dina Nair, Joel S. Klinton, et al. Pre-diagnosis attrition but high pre-treatment attrition among patients with MDR-TB: An operational research from Chennai,India. *J Epidemiol Glob Health*. 2017;7(4):227-233.
13. Boyd R, Ford N, Padgen P, Cox H. Time to treatment for rifampicin-resistant tuberculosis: Systematic review and meta-analysis. *Int J Tuberc Lung Dis.* 2017;21(11):1173-1180. doi:10.5588/ijtld.17.0230
14. Xu C, Li R, Shewade HD, et al. Attrition and delays before treatment initiation among patients with MDR-TB in China (2006-13): Magnitude and risk factors. *PLoS One*. 2019;14(4):1-16. doi:10.1371/journal.pone.0214943
15. Hirasen K, Berhanu R, Evans D, et al. High rates of death and loss to follow-up by 12 months of rifampicin resistant TB treatment in South Africa. *PLoS One*. 2018;13(10):1-14. doi:10.1371/journal.pone.0205463
16. Shewade D, Kokane AM, Singh AR, et al. High pre-diagnosis attrition among patients with presumptive MDR-TB: an operational research from Bhopal district, India. *BMC Health Serv Res*. 2017;17(1):1-10. doi:10.1186/s12913-017-2191-6
17. Oo T, Kyaw KWY, Soe KT, et al. Magnitude and reasons for pre-diagnosis attrition among presumptive multi-drug resistant tuberculosis patients in Bago Region, Myanmar: A mixed methods study. *Sci Rep*. 2019;9(1):1-10. doi:10.1038/s41598-019-43562-3
18. Xu C, Pang Y, Li R, et al. Clinical outcome of multidrug-resistant tuberculosis patients receiving standardized second-line treatment regimen in China. *J Infect.* 2018;76(4):348-353. doi:10.1016/j.jinf.2017.12.017
19. Abubakar I, Zignol M, Falzon D, et al. Drug-resistant tuberculosis: Time for visionary political leadership. *Lancet Infect Dis.* 2013;13(6):529-539. doi:10.1016/S1473-3099(13)70030-6
20. Radin E, Ariana P, Broekel T et al. Analyzing demand-side efficiency in global health: an application to maternal care in Vietnam. *Health Policy Plan.* 2016;31(9):1281-1290. doi:10.1093/heapol/czw063
21. Boehme CC, Nicol MP, Nabeta P, et al. Feasibility, diagnostic accuracy, and effectiveness of decentralised use of the Xpert MTB/RIF test for diagnosis of tuberculosis and multidrug resistance: A multicentre implementation study. *Lancet.* 2011;377(9776):1495-1505. doi:10.1016/S0140-6736(11)60438-8
22. Christopher PM, Cucunawangsih, Widysanto A. GeneXpert Mycobacterium tuberculosis/ rifampicin assay for molecular epidemiology of rifampicin-resistant Mycobacterium tuberculosis in an urban setting of Banten province, Indonesia. *Int J Mycobacteriol* 2019;8:351-8.
23. Atashi S, Izadi B, Jalilian S. et al. Evaluation of GeneXpert MTB / RIF for determination of rifampicin resistance among new tuberculosis cases in west and northwest Iran. *New Microbes New Infect*. 2017;19:117-120. doi:10.1016/j.nmni.2017.07.002
24. Pang, Y. , Xia, H. , Zhang, Z., et al. Multicenter evaluation of genechip for detection of multidrug-resistant mycobacterium tuberculosis. *Journal of Clinical Microbiology*, 51(6), 1707-1713. 2013;51(6):1707-1713. doi:10.1128/JCM.03436-12
25. Yin J, Yuan J, Hu Y, Wei X. Association between directly observed therapy and treatment



For peer review only - http://bmjopen.bmj.com/site/about/guidelines.xhtml

outcomes in multidrug-resistant tuberculosis: A systematic review and meta-analysis. *PLoS One*. 2016;11(3):1-14. doi:10.1371/journal.pone.0150511

26. Narges A , Leah J , Cecily M , et al. Adherence interventions and outcomes of tuberculosis treatment: A systematic review and meta-analysis of trials and observational studies. *PLoS Med*, 2018, 15(7):e1002595. doi:10.1371/journal.pmed.1002595
27. Baral SC, Aryal Y, Bhattrai R, et al. The importance of providing counselling and financial support to patients receiving treatment for multi-drug resistant TB: Mixed method qualitative and pilot intervention studies. *BMC Public Health.* 2014;14(1). doi:10.1186/1471-2458-14-46
28. Wang L, Li R, Xu C, et al. The Global Fund in China: Multidrug-resistant tuberculosis nationwide programmatic scale-up and challenges to transition to full country ownership. *PLoS One*. 2017;12(6):1-13. doi:10.1371/journal.pone.0177536
29. Subbaraman R, Nathavitharana RR, Mayer KH, et al. Constructing care cascades for active tuberculosis: A strategy for program monitoring and identifying gaps in quality of care. *PLoS Med*. 2019;16(2):1-18. doi:10.1371/journal.pmed.1002754
30. China National Bureau of Statistics. Accessed August, 2020 http://www.stats.gov.cn/tjsj/ndsj/2019/indexch.htm
31. Truzyan N, Crape B, Grigoryan R, et al. Increased risk for multidrug-resistant tuberculosis in migratory workers, Armenia. *Emerg Infect Dis.* 2015;21(3):474-476. doi:10.3201/eid2103.140474
32. Parmar MM, Sachdeva KS, Dewan PK, et al. Unacceptable treatment outcomes and associated factors among India's initial cohorts of multidrug-resistant tuberculosis (MDR-TB) patients under the revised national TB control programme (2007–2011): Evidence leading to policy enhancement. *PLoS One*. 2018;13(4):1-24. doi:10.1371/journal.pone.0193903
33. Lalor MK, Greig J, Allamuratova S, et al. Risk factors associated with default from multi- and extensively drug-resistant tuberculosis treatment, Uzbekistan: A retrospective cohort analysis. *PLoS One*. 2013;8(11). doi:10.1371/journal.pone.0078364
34. Li X, Yang Q, Feng B, et al. Tuberculosis infection in rural labor migrants in Shenzhen, China: Emerging challenge to tuberculosis control during urbanization. *Sci Rep*. 2017;7(1):1-8. doi:10.1038/s41598-017-04788-1
35. Zumla A, Abubakar I. Improving access to multi-drug resistant tuberculosis diagnostic and health services for refugees and migrants. *BMC Med*. 2018;16(1):7-10. doi:10.1186/s12916-018-1218-0
36. Wang W, Wang J, Zhao Q, et al. Contribution of rural-to-urban migration in the prevalence of drug resistant tuberculosis in China. Eur J Clin Microbiol Infect Dis. 2011;30(4):581-586. doi:10.1007/s10096-010-1125-6
37. Nellums LB, Rustage K, Hargreaves S, et a;. Multidrug-resistant tuberculosis treatment adherence in migrants: A systematic review and meta-analysis. *BMC Me*d. 2018;16(1):1-11. doi:10.1186/s12916-017-1001-7
38. Long Q, Li Y, Wang Y, et al. Barriers to accessing TB diagnosis for rural-to-urban migrants with chronic cough in Chongqing, China: A mixed methods study. *BMC Health Serv Res*. 2008;8:1-10. doi:10.1186/1472-6963-8-202
39. Wei X, Chen J, Chen P, et al. Barriers to TB care for rural-to-urban migrant TB patients in Shanghai: A qualitative study. *Trop Med Int Heal.* 2009;14(7):754-760. doi:10.1111/j.1365-3156.2009.02286.x



For peer review only - http://bmjopen.bmj.com/site/about/guidelines.xhtml

40. Wei X, Zou G, Yin J, et al. Providing financial incentives to rural-to-urban tuberculosis migrants in Shanghai: An intervention study. *Infect Dis Poverty.* 2012;1(1):1-8. doi:10.1186/2049-9957-1-9

41. Zhejiang Bureau of Statistics. Statistical Communique on the provincial economy and social development of Zhejiang in 2015 and 2018, Accessed February, 2021. http://tjj.zj.gov.cn/col/col1229129205/index.html

**FIGURES AND LEGENDS**

Figure 1: Seven steps of the DRTB diagnosis and care cascade

Figure 2: Characteristics of diagnosed, registered and treated RR/MDR-TB patients, 2015-2018

Figure 3: Combination of DRTB policies implemented in each prefecture from 2015-2018

For peer review only



For peer review only - http://bmjopen.bmj.com/site/about/guidelines.xhtml

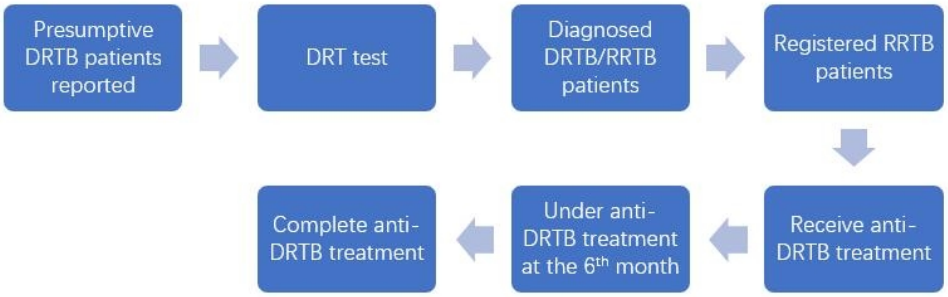


210x71mm (96 x 96 DPI)

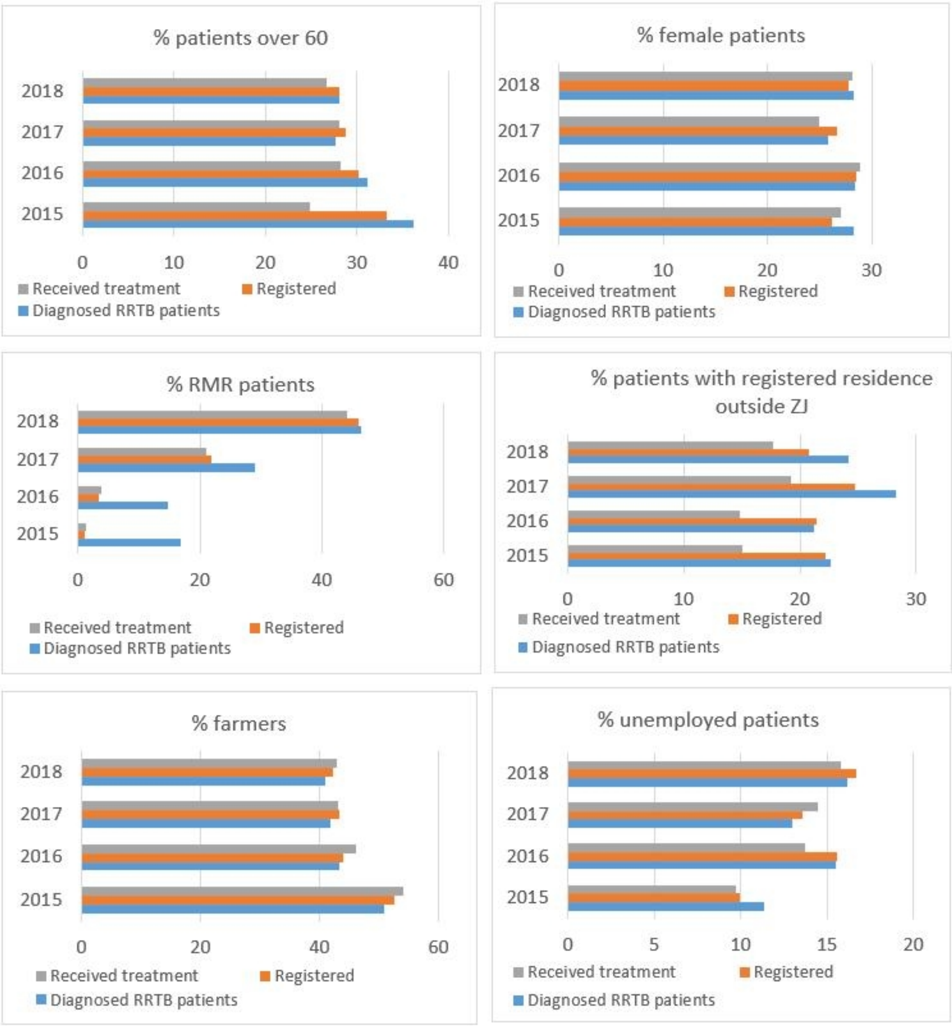


206x217mm (96 x 96 DPI)

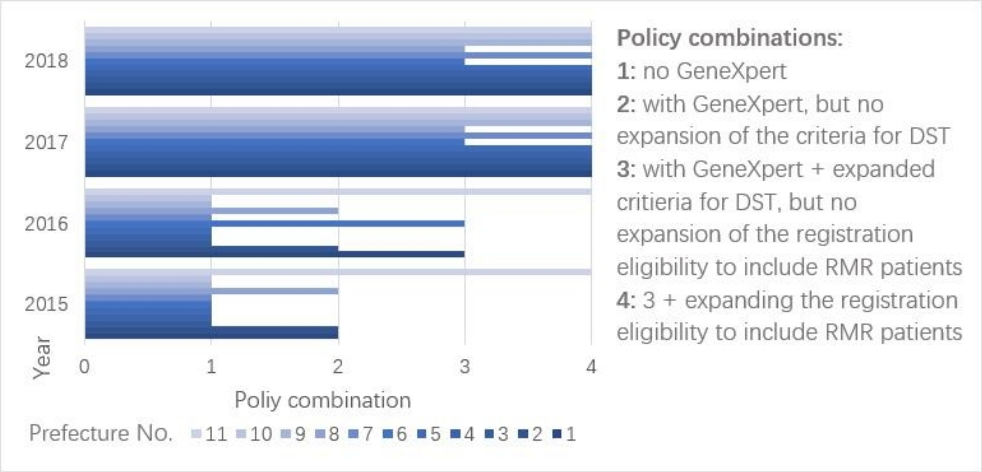


207x102mm (96 x 96 DPI)

## 1. Policy survey questionnaire

### Drug-resistant tuberculosis related policy survey in Zhejiang

Prefecture: __________

Institute: __________

Date: _______(YY/MM/DD)

1. Eligibility criteria for drug-resistant tuberculosis (DRTB) patient screening
    1) The current eligibility criteria of patients referred for screening are: ________________________________________________________________________
    2) Are there any changes in the eligibility criteria after 2014: _____yes/no
       If yes: year of change_______, and the eligibility criteria for patients referred for screening before policy change_______________________________________

(If the eligibility criterion is the "high-risk" TB patients, please specify the definition of high-risk TB patients in your prefecture, for example, smear positive after two months' treatment or others).

2. Test tools for drug susceptibility testing (DST)
    1) Has GeneXpert been equipped in the prefecture-level designated hospital? If yes, it was equipped in the year ______
    2) Under what conditions would patients be referred for traditional DRTB tests after screening? ___________________________
    3) Please list other types of DST conducted in the prefecture, including both molecular and traditional________.
    4) Does the government provide funds to secure the supply of the reagents for rapid DST now? _______(Yes/no). If yes, at which year did the government begin to provide such funds? ____.
3. Financing of DST
    1) Does the patient need to pay out-of-pocket for the DRTB rapid screening test now? _____ (yes/no). If yes, how much does the patient need to pay? RMB_____, accounting for _____% of the total cost
    2) Does the government pay for the DRTB rapid screening test now? (yes/no). If yes, how much does the government pay for each test? RMB_____, accounting for _____% of the total cost
    3) Does the health insurance pay for the DRTB rapid screening test now? ______ (yes/no). If yes, _____% of the cost would be reimbursed by the health insurance.
    4) Does the health insurance pay for the DRTB rapid screening test now? (yes/no). If yes, _____% of the cost would be reimbursed by the health insurance.
    5) Have the polices on the financing of DST ever been changed after 2014? If yes, the year of change is ______. Before the policy change,
       the patient paid for RMB____ for the screening test, accounting for _____% of the total cost;
       The government paid for _____% of the total cost;

the health insurance schemes reimbursed ________% of the total cost;

other funding resources paid for _______% of the total cost

4. DRTB patient registration

   1) Currently what types of DRTB patients would be registered and managed in the TB information management system (TBIMS)? (Please check all that apply)

      a. XDR and MDRTB b. rifampin mono-resistant (RMR) patients c. Mono-resistant to other type of first-line drug

   2) Has this policy been changed after 2014? _____(yes/no). If yes, please specify the types of DRTB patients that were registered and under management before policy change. (please check all that apply)

      a. XDR and MDRTB b. rifampin mono-resistant (RMR) patients c. Mono-resistant to other type of first-line drugs

5. DRTB treatment

   1) The standard anti-DRTB treatment regimen includes ______ months' hospitalization, and the total treatment length is _____months

   2) Health insurance policies:

      a) Has the specialized outpatient reimbursement program for DRTB treatment been implemented in your prefecture now? _______(yes/no) If so, this policy began in the year_______. The reimbursement rate for outpatient service is ______%, and the annual limit line for compensation is RMB_______.

      b) The reimbursement rate for DRTB inpatient service is ______%, the deductible is RMB_____, and the annual limit line for compensation is RMB_______.

      c) Has DRTB treatment been included in the serious disease reimbursement program of the health insurance? ________(yes/no). If yes, this program started in the year________, for the out-of-pocket payment over RMB_________, the reimbursement rate is ______%, and the annual limit line is RMB________.

      d) Could RMR patients enjoy the health insurance policies for anti-DRTB treatment now? ________(yes/no). If yes, at which year could the RMR patients start to enjoy such policies? _______.

      e) Are these health insurance policies only eligible for those who enrolled in the local health insurance schemes? _______(yes/no). If no, at which year could patients with health insurance outside your prefecture/Zhejiang province start to enjoy such policies? _______.

      f) Could the health insurance policies be enjoyed if patients do not get cured after the standard treatment length? ______(yes/no). If yes, how long could patients enjoy these policies? _______________________________________________________.

   3) Other government financial assistance policies for DRTB patients

      a) Are there any other financial assistance policies for DRTB patients in your prefecture? _________(yes/no). If yes, at which year were they launched? Please briefly describe the policy? (How much money given to DRTB patients, how to deliver such assistance, etc.) ______________________________________

      b) Could RMR patients enjoy the financial assistance policies for now? _______(yes/no). If yes, at which year could the RMR patients start to enjoy

For peer review only

such policies? _______.

c) Could patients without local registered residence (Hukou) enjoy the policy now? ________(yes/no). If yes, at which year could the RMR patients without local Hukou enjoy such policies? __________.

d) How long could the patients enjoy these policies after diagnosis? ________

6. Any other DRTB related policies, please describe here________________

## 2. Supplementary result tables

Table 1: Year of policy change regarding the screening test and registration in the 11 prefectures

| Prefecture No. | Year of policy change | | |
|---|---|---|---|
| | screening range | test technology | registration policy |
| 1 | 2016 | 2015 | 2017 |
| 2 | 2017 | 2014 | 2014 |
| 3 | 2017 | 2017 | 2017 |
| 4 | 2017 | 2017 | 2017 |
| 5 | 2017 | 2017 | 2017 |
| 6 | 2009 | 2016 | 2019 |
| 7 | 2017 | 2017 | 2017 |
| 8 | 2017 | 2015 | 2019 |
| 9 | 2017 | 2017 | 2017 |
| 10 | 2017 | 2017 | 2015 |
| 11 | 2000 | 2012 | 2012 |

Table 2: Starting time and eligibility for health insurance and medical assistance policy coverage, 2015-2018

| Prefecture | Health insurance policy | | | Medical assistance | | |
|---|---|---|---|---|---|---|
| No. | starting time | insurance outside ZJ | RMR | starting time | registered residence outside ZJ | RMR |
| 1 | 2018 | N | Y | 2013 | N | N |
| 2 | 2013 | N | Y | 2013 | Y | Y since 2015 |
| 3 | 2016 | N | N | 2014 | Y | N |
| 4 | 2017 | N | Y | 2015 | Y | N |
| 5 | 2016 | N | N | 2015 | N | N |
| 6 | 2019 | N | N | 2012 | N | Y |
| 7 | 2014 | N | N | 2014 | Y | N |
| 8 | 2013 | N | N | 2015 | N | Y |
| 9 | 2014 | N | Y since 2017 | No policy | | |
| 10 | 2013 | N | Y | 2015 | N | Y |
| 11 | 2011 | N | Y | 2015 | Y | Y |

Table 3: The diagnostic test for presumptive DRTB patients from 2015-2018

| Year | No. of | % took | % took | % took | Patients with no DST |
|---|---|---|---|---|---|

For peer review only

| | presumptive patients reported | fast DST | traditional DST | both tests | total | records (%) not TB | unknown reason |
|---|---|---|---|---|---|---|---|
| 2015 | 9285 | 18.2 | 51.3 | 0.2 | 30.7 | 13.4 | 17.3 |
| 2016 | 10997 | 30.4 | 46.9 | 0.5 | 23.3 | 11.6 | 11.7 |
| 2017 | 21768 | 30.9 | 42.8 | 5.8 | 32.2 | 25.6 | 6.6 |
| 2018 | 23916 | 64.4 | 33.8 | 20.1 | 21.9 | 19.3 | 2.7 |

Table 4: Reasons for not completing anti-DRTB treatment according to the registration records

| Year | Total | Under treatment | | Dead | | lost-to-follow-up | | Other reasons | |
|---|---|---|---|---|---|---|---|---|---|
| | | No. | % | No. | % | No. | % | No. | % |
| 2015 | 68 | 11 | 16.2 | 17 | 25.0 | 13 | 19.1 | 27 | 39.7 |
| 2016 | 101 | 38 | 37.6 | 15 | 14.9 | 15 | 14.9 | 33 | 32.7 |

For peer review only

STROBE Statement—Checklist of items that should be included in reports of ***cross-sectional studies***

| | Item No | Recommendation | Page No |
|---|---|---|---|
| **Title and abstract** | 1 | (*a*) Indicate the study's design with a commonly used term in the title or the abstract | 1 |
| | | (*b*) Provide in the abstract an informative and balanced summary of what was done and what was found | 3 |
| **Introduction** | | | |
| Background/rationale | 2 | Explain the scientific background and rationale for the investigation being reported | 5 |
| Objectives | 3 | State specific objectives, including any prespecified hypotheses | 6 |
| **Methods** | | | |
| Study design | 4 | Present key elements of study design early in the paper | 7 |
| Setting | 5 | Describe the setting, locations, and relevant dates, including periods of recruitment, exposure, follow-up, and data collection | 7,8 |
| Participants | 6 | (*a*) Give the eligibility criteria, and the sources and methods of selection of participants | 8 |
| Variables | 7 | Clearly define all outcomes, exposures, predictors, potential confounders, and effect modifiers. Give diagnostic criteria, if applicable | NA |
| Data sources/ measurement | 8* | For each variable of interest, give sources of data and details of methods of assessment (measurement). Describe comparability of assessment methods if there is more than one group | *8* |
| Bias | 9 | Describe any efforts to address potential sources of bias | NA |
| Study size | 10 | Explain how the study size was arrived at | 8 |
| Quantitative variables | 11 | Explain how quantitative variables were handled in the analyses. If applicable, describe which groupings were chosen and why | 10 |
| Statistical methods | 12 | (*a*) Describe all statistical methods, including those used to control for confounding | 10 |
| | | (*b*) Describe any methods used to examine subgroups and interactions | 10 |
| | | (*c*) Explain how missing data were addressed | NA |
| | | (*d*) If applicable, describe analytical methods taking account of sampling strategy | NA |
| | | (*e*) Describe any sensitivity analyses | NA |
| **Results** | | | |
| Participants | 13* | (a) Report numbers of individuals at each stage of study—eg numbers potentially eligible, examined for eligibility, confirmed eligible, included in the study, completing follow-up, and analysed | 12,13 |
| | | (b) Give reasons for non-participation at each stage | NA |
| | | (c) Consider use of a flow diagram | NA |
| Descriptive data | 14* | (a) Give characteristics of study participants (eg demographic, clinical, social) and information on exposures and potential confounders | 14 |
| | | (b) Indicate number of participants with missing data for each variable of interest | NA |
| Outcome data | 15* | Report numbers of outcome events or summary measures | 12,13 |
| Main results | 16 | (*a*) Give unadjusted estimates and, if applicable, confounder-adjusted estimates and their precision (eg, 95% confidence interval). Make clear which confounders were adjusted for and why they were included | 15,16, 17,18 |

For peer review only

| | | | |
|---|---|---|---|
| | | (*b*) Report category boundaries when continuous variables were categorized | 10 |
| | | (*c*) If relevant, consider translating estimates of relative risk into absolute risk for a meaningful time period | NA |
| Other analyses | 17 | Report other analyses done—eg analyses of subgroups and interactions, and sensitivity analyses | NA |
| **Discussion** | | | |
| Key results | 18 | Summarise key results with reference to study objectives | 18 |
| Limitations | 19 | Discuss limitations of the study, taking into account sources of potential bias or imprecision. Discuss both direction and magnitude of any potential bias | 20 |
| Interpretation | 20 | Give a cautious overall interpretation of results considering objectives, limitations, multiplicity of analyses, results from similar studies, and other relevant evidence | 18,19 20 |
| Generalisability | 21 | Discuss the generalisability (external validity) of the study results | 20 |
| **Other information** | | | |
| Funding | 22 | Give the source of funding and the role of the funders for the present study and, if applicable, for the original study on which the present article is based | 21 |

*Give information separately for exposed and unexposed groups.

**Note:** An Explanation and Elaboration article discusses each checklist item and gives methodological background and published examples of transparent reporting. The STROBE checklist is best used in conjunction with this article (freely available on the Web sites of PLoS Medicine at http://www.plosmedicine.org/, Annals of Internal Medicine at http://www.annals.org/, and Epidemiology at http://www.epidem.com/). Information on the STROBE Initiative is available at www.strobe-statement.org.

For peer review only



For peer review only - http://bmjopen.bmj.com/site/about/guidelines.xhtml
